# β-Catenin C-terminal signals suppress p53 and are essential for artery formation

**DOI:** 10.1038/ncomms12389

**Published:** 2016-08-08

**Authors:** Dario F. Riascos-Bernal, Prameladevi Chinnasamy, Longyue (Lily) Cao, Charlene M. Dunaway, Tomas Valenta, Konrad Basler, Nicholas E. S. Sibinga

**Affiliations:** 1 Department of Medicine (Cardiology Division), Department of Developmental and Molecular Biology, and Wilf Family Cardiovascular Research Institute, Albert Einstein College of Medicine, 1300 Morris Park Avenue, Bronx, New York 10461, USA; 2 Institute of Molecular Life Sciences, University of Zurich, Winterthurerstrasse 190, Zurich, CH-8057, Switzerland

## Abstract

Increased activity of the tumour suppressor p53 is incompatible with embryogenesis, but how p53 is controlled is not fully understood. Differential requirements for p53 inhibitors Mdm2 and Mdm4 during development suggest that these control mechanisms are context-dependent. Artery formation requires investment of nascent endothelial tubes by smooth muscle cells (SMCs). Here, we find that embryos lacking SMC β-catenin suffer impaired arterial maturation and die by E12.5, with increased vascular wall p53 activity. β-Catenin-deficient SMCs show no change in p53 levels, but greater p53 acetylation and activity, plus impaired growth and survival. *In vivo*, SMC p53 inactivation suppresses phenotypes caused by loss of β-catenin. Mechanistically, β-catenin C-terminal interactions inhibit Creb-binding protein-dependent p53 acetylation and p53 transcriptional activity, and are required for artery formation. Thus in SMCs, the β-catenin C-terminus indirectly represses p53, and this function is essential for embryogenesis. These findings have implications for angiogenesis, tissue engineering and vascular disease.

## Abstract (web summary)

How p53 is restrained in arterial maturation during embryonic development is unclear. Here, the authors show that β-catenin C-terminal interactions inhibit CREB binding protein-mediated acetylation and activation of p53 in smooth muscle cells, and that this function is essential for artery formation.

## Graphical abstract

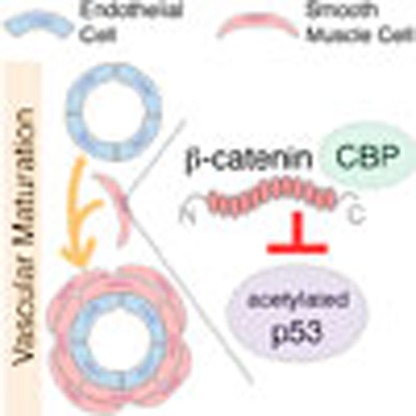

The p53 tumour suppressor plays a fundamental role in cell biology by inducing cell cycle arrest and apoptosis. Increased p53 activity is incompatible with normal embryogenesis, a process characterized by active growth. Indeed, inactivation of the key inhibitors of p53, Mdm2 and Mdm4, causes embryonic lethality^1^,^2^, and increased p53 activity during development yields a phenotype resembling CHARGE syndrome^3^. Although Mdm2 and Mdm4 are the major p53 inhibitors^4^, the differential requirement for Mdm2 and Mdm4 in cardiac and central nervous system development^5^ raises the possibility that cell type-specific mechanisms restrain p53 during embryogenesis. This is especially intriguing during artery formation: although smooth muscle cells (SMCs) constitute the main component of the arterial wall, requirements for Mdm2 or Mdm4 have not been evaluated in this cell type, and how SMCs repress p53 function to proliferate, survive and build the arterial wall is unknown.

Development of a competent vasculature reflects the integration of several processes, including vasculogenesis, angiogenesis, remodelling and vascular maturation. Despite recent advances in this area^6^, understanding of vascular maturation remains limited. Mural cells—SMCs in large vessels or pericytes in the microcirculation—surround nascent endothelial tubes in a process called investment^7^ to provide quiescence and stability^8^, and proliferate and synthesize extracellular matrix components to build a multilayered wall^9^, but how these activities are regulated is only partly understood.

β-Catenin, the non-redundant component of the canonical Wnt signalling pathway and structural element of the cadherin–catenin cell-adhesion complex^10^, has critical functions in embryogenesis and adult homoeostasis^11^. In the vasculature, endothelial cell β-catenin is required for angiogenesis in the central nervous system and contributes to endothelial arterial specification^12^,^13^. β-Catenin is also necessary for smooth muscle development in the lung^14^ and in the proepicardium for coronary artery formation^15^. While β-catenin promotes proliferation and survival of cultured SMCs, and its expression and activity are induced in adult arteries during vascular remodelling^16^,^17^,^18^,^19^, whether it is required for vascular maturation during development is unknown.

To address this question, we inactivated β-catenin selectively in SMCs and tested a series of β-catenin mutants *in vivo*, and found an absolute requirement for SMC β-catenin in artery formation and mouse embryogenesis. Here, we provide evidence that β-catenin signalling in SMCs is essential for artery formation. Loss of this activity, particularly that emanating from the β-catenin C-terminal interaction domain, results in an incompetent circulation that cannot support embryogenesis. Interestingly, β-catenin performs this function in part by inhibiting p53 activity.

## Results

### SMC β-catenin is essential for artery formation

**Figure 1:**
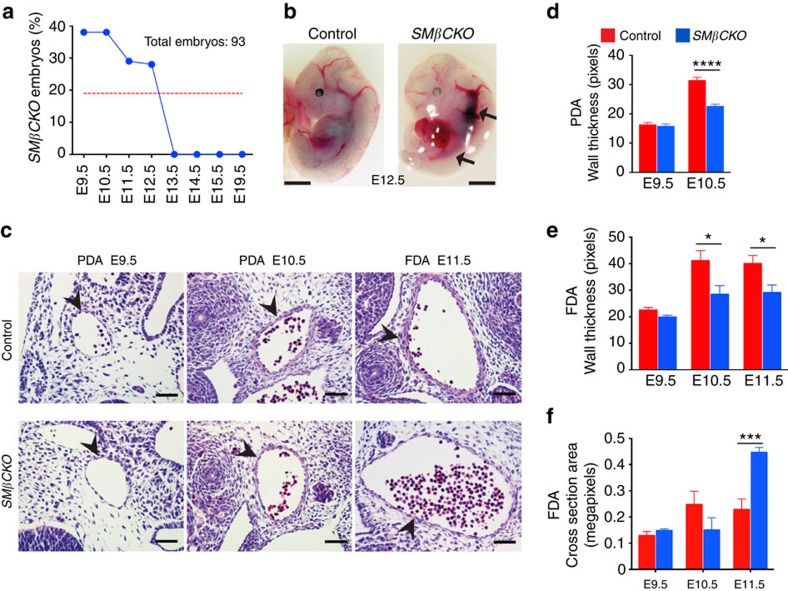
SMC β-catenin is essential for embryonic survival and artery formation. (**a**) Observed frequency of *SMβCKO* embryos. The red line indicates the expected frequency. No *SMβCKO* were found at embryonic stage (E) 13.5 or beyond. (**b**) Mouse embryos at E 12.5. Arrows indicate enlarged blood vessels. Scale bar, 1 mm (**c**) Hematoxylin and eosin-stained sections of the PDA and FDA. Arrowheads indicate the vessel wall. Scale bar, 50 μm. (**d**,**e**) Quantification of wall thickness of the PDA (*n*=8 in every group) and FDA (*n*=4 in every group). ****P<0.0001; *P<0.05. (**f**) Quantification of the cross-section area of the FDA (*n*=4 in every group). ***P<0.001. In **d**–**f**, data represent the mean ±s.e.m., groups were compared by two-way ANOVA and Bonferroni's multiple comparisons test.

We studied SMC-selective inactivation of *Ctnnb1 (β-catenin)* by crossing *Tagln-Cre* transgenic^20^ and *Ctnnb1*^*flox/flox*^ mice^21^. We found no surviving *Tagln-Cre;Ctnnb1*^*flox/flox*^ (*SMβCKO*; see Table 1 for mouse line abbreviations) progeny at E13.5 or beyond (Fig. 1a; Supplementary Tables 1 and 2; Supplementary Fig. 1a,b). At E12.5, all control embryos looked normal, while all *SMβCKO* embryos were compromised, ranging in appearance from early stages—small size, dilated blood vessels and compromised overall development, but retaining recognizable morphologic features (Fig. 1b)—to advanced stages of resorption—still smaller, generally round and pale in appearance, and lacking morphological features. Mutant embryos appeared grossly normal until E10.5, indicating that vasculogenesis and early angiogenesis were largely unaffected. Evaluation of *SMβCKO* embryos revealed reduced wall thickness of the paired and fused dorsal aortas (PDA and FDA, respectively) as early as E10.5 (Fig. 1c–e), plus increased cross-sectional area of the FDA at E11.5 (Fig. 1c,f); immunohistochemistry confirmed decreased β-catenin expression in the vessel wall and heart (Supplementary Fig. 1c). Because *Tagln* is transiently expressed in the heart, we also looked for a potential cardiac phenotype, but did not find (1) signs of cardiac dysfunction, such as oedema or pericardial effusion (Supplementary Fig. 1d), (2) differences in the future left or right ventricles, compact or trabeculated myocardium (Supplementary Fig. 1e–g), (3) change in outflow tract (OT) dimensions in *SMβCKO* embryos at E10.5 (Supplementary Fig. 1h) or (4) major structural abnormalities at E11.5 (Supplementary Fig. 1i)—these observations differ from a report of β-catenin inactivation using a different *Tagln-Cre* transgenic line, which describes severe right ventricular hypoplasia at E9.5 and demise between E10.5 and E11.5 (ref. 22). In our system, possibly due to different transgene integration site or modest variation in level, timing or distribution of *Tagln-Cre* expression or strain background, we observe that β-catenin loss in SMCs impairs artery formation, as reflected by thinned and dilated major vessels observed before any evident cardiac abnormality.

### β-catenin promotes arterial SMC proliferation and survival

**Figure 2:**
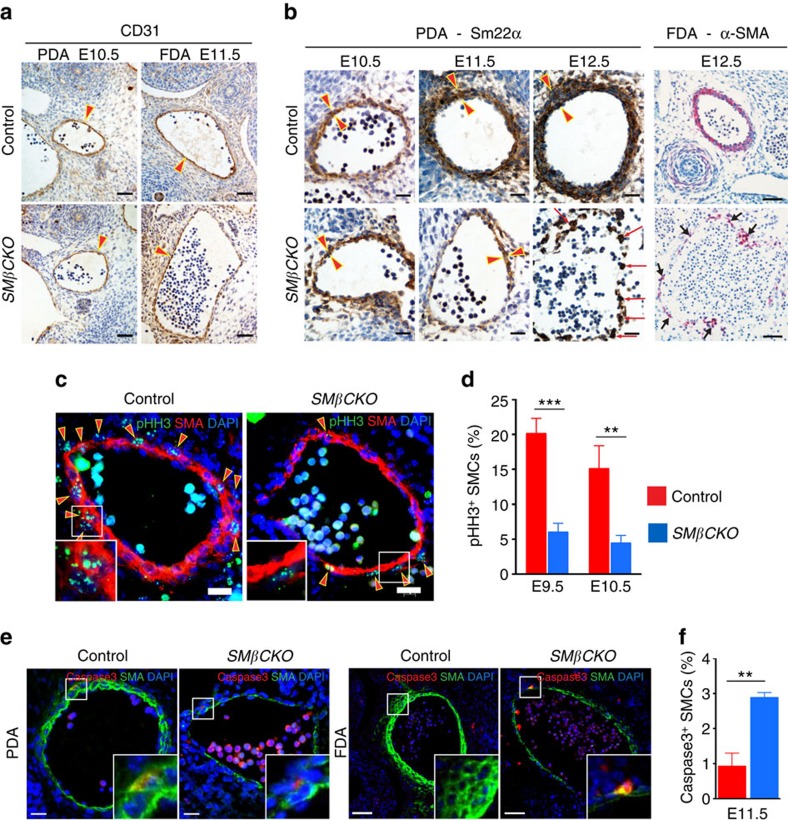
β-Catenin promotes SMC proliferation and survival during artery formation. (**a**) Immunohistochemistry (IHC) for the endothelial marker CD31. Arrowheads indicate the endothelial layer. Scale bar, 50 μm. (**b**) IHC for SMC markers, Sm22α (brown; scale bar, 25 μm) and α-SMA (red; scale bar, 50 μm). Arrowheads delimit the vessel wall. Arrows indicate scattered SMCs. (**c**) Immunostaining of PDAs for the mitotic marker pHH3 and α-SMA at E10.5. Arrowheads indicate pHH3^+^ SMCs. Scale bar, 20 μm. (**d**) Quantification of pHH3^+^ SMCs in the wall of PDAs. **P<0.01; ***P<0.001 comparing genotypes by two-way ANOVA and Sidak's multiple comparison test. *n*=6. (**e**) Immunostaining for cleaved Caspase3 and α-SMA at E11.5. Scale bar, 20 μm (PDA); 50 μm (FDA). (**f**) Quantification of Caspase3^+^ SMCs in the arterial wall. **P<0.01 comparing genotypes by two-tailed *t*-test. *n*=3. In **d**,**f**, data represent the mean±s.e.m.

The arterial wall has two main cellular components: endothelial cells and SMCs. While CD31 staining demonstrated an intact endothelial monolayer in both control and *SMβCKO* embryos (Fig. 2a; Supplementary Fig. 2a), staining for SMC markers showed lack of SMC investment and absence of a multilayered wall by E12.5 in *SMβCKO* embryos (Fig. 2b). We examined SMC-specific proliferation in the PDA wall by co-staining two markers of proliferation, phospho-histone H3 (pHH3) or Ki67, with smooth muscle α-actin (α-SMA), and found reduced SMC proliferation in *SMβCKOs* at E9.5 and E10.5 (Fig. 2c,d; Supplementary Fig. 2b). We also observed decreased cell proliferation in the FDA wall from E9.5 to E11.5 (Supplementary Fig. 2c). In addition, by both TUNEL assay and co-staining of cleaved Caspase 3 and α-SMA, we observed increased SMC apoptosis in the *SMβCKO* arterial wall (Fig. 2e,f; Supplementary Fig. 2d). These findings suggest that the necessity for SMC β-catenin in formation of a multilayered arterial wall stems from its positive effects on cell proliferation and survival.

### β-catenin is required for SMC population growth

**Figure 3:**
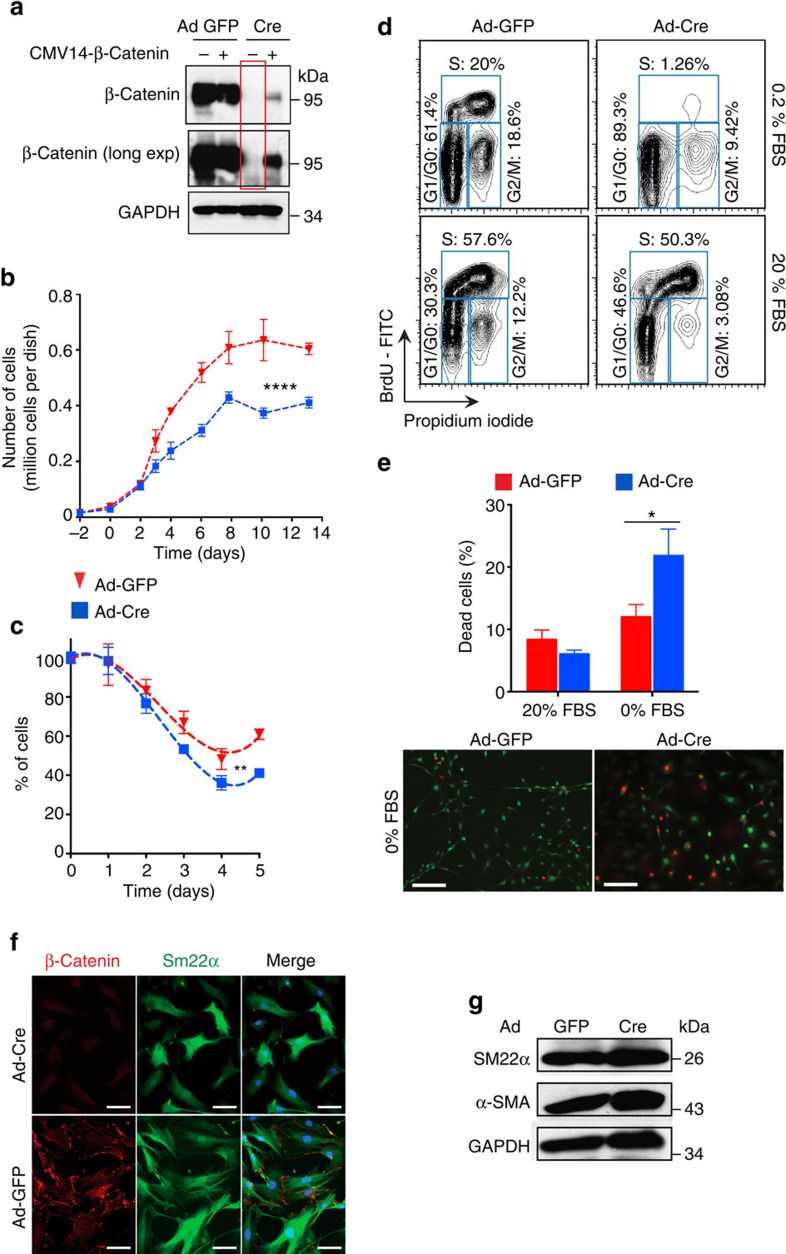
β-Catenin is required for vascular SMC population growth. (**a**) Western blot analysis of indicated proteins in mouse aortic SMCs isolated from *Ctnnb1*^*flox/flox*^ mice, transduced with GFP- or Cre-expressing adenovirus (Ad-GFP or Ad-Cre) and transfected with CMV14-β-catenin or empty vector. β-Catenin expression is not detectable in Ad-Cre SMCs even with a long exposure (red box). β-Catenin expression restored by lipid-based transfection of CMV14-β-catenin in Ad-Cre SMCs (fourth lane). (**b**) Mouse aortic SMC population growth under standard growth conditions. ****P<0.0001 comparing Ad-GFP versus Ad-Cre by two-way ANOVA. (**c**) Mouse aortic SMC population decline under serum starvation. **P<0.01 comparing Ad-GFP versus Ad-Cre by two-way ANOVA. (**d**) FACS analysis of BrdU and propidium iodide staining in mouse aortic SMCs with the indicated treatments and in both low and high serum conditions. (**e**) Top: quantification of dead cells. *P<0.05 comparing Ad-GFP versus Ad-Cre by two-way ANOVA and Sidak's multiple comparisons test. Bottom: LIVE/DEAD assay using mouse aortic SMCs with the indicated treatments. Live cells in green; dead cells in red. Scale bar, 200 μm. (**f**) Immunocytochemistry, scale bar, 50 μm and (**g**) western blot analysis of β-catenin and indicated SMC markers in Ad-GFP or Ad-Cre-transduced mouse aortic SMCs. In **b**,**c**,**e**, data represent the mean±s.e.m. and *n*=3.

To evaluate how β-catenin affects basic cell functions, we isolated aortic SMCs from *Ctnnb1*^*flox/flox*^ mice and transduced them with Cre- or GFP-expressing adenovirus to obtain β-catenin-deficient and control SMCs, respectively (Fig. 3a). In culture, SMCs lacking β-catenin grew more slowly and plateaued at a lower density (Fig. 3b). Moreover, with serum starvation, the β-catenin-deficient cell population decreased faster and to a greater extent (Fig. 3c). Cell cycle analyses found more β-catenin-deficient SMCs in the G_0_/G_1_ phase and less in S or G_2_/M (Fig. 3d), indicating that β-catenin is required for cell cycle progression. Serum deprivation caused more death of β-catenin-deficient than control cells (Fig. 3e), showing that SMC β-catenin is a pro-survival factor. On the other hand, SMC marker gene expression and transwell cell migration were not affected by β-catenin loss, suggesting a lack of effect on maintenance of SMC differentiation and motility (Fig. 3f,g; Supplementary Fig. 3).

### β-catenin opposes p53 acetylation and activity in SMCs

**Figure 4:**
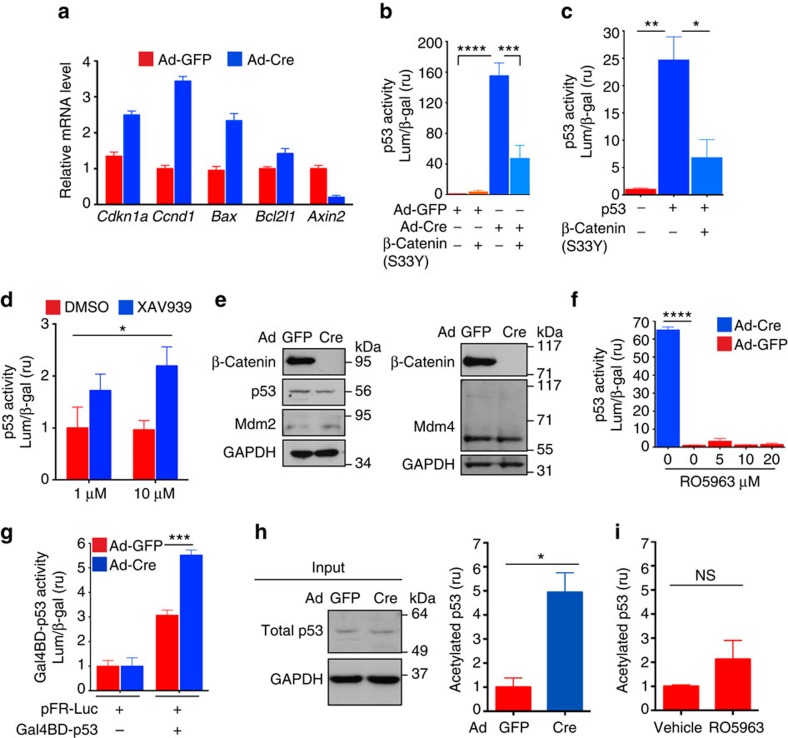
β-Catenin inhibits p53 acetylation and activity in vascular SMCs. (**a**) RT-qPCR of indicated genes in mouse aortic SMCs, Ad-GFP (control) versus Ad-Cre (β-catenin-deficient), normalized to *Rps13*. Data represent the mean ±s.d., *n*=3. (**b**) p53 activity was measured with the p53 reporter, PG13-Luc plasmid, in mouse aortic SMCs. β-Catenin (S33Y), constitutively active β-catenin. Luminescence (Lum) was normalized to β-galactosidase activity (β-gal), to control for transfection efficiency. ***P<0.001; ****P<0.0001. (**c**) p53 activity in mouse aortic SMCs electroporated with PG13-Luc and the indicated expression vectors. *P<0.05; **P<0.01. (**d**) p53 activity in mouse aortic SMCs electroporated with PG13-Luc and treated with β-catenin inhibitor XAV939 or vehicle (DMSO). *P<0.05 comparing DMSO versus XAV939 by two-way ANOVA. (**e**) Western blot analysis of the indicated proteins in mouse aortic SMCs, Ad-GFP versus Ad-Cre. (**f**) p53 activity in mouse aortic SMCs electroporated with PG13-Luc, and exposed to increasing concentrations of RO5963, an Mdm2/Mdm4 inhibitor. ****P<0.0001. (**g**) Gal4BD-p53 transcriptional activity in mouse aortic SMCs electroporated with pFR-Luc and Gal4BD-p53 as indicated. ***P<0.001 comparing Ad-GFP versus Ad-Cre by two-way ANOVA and Sidak's multiple comparisons test. (**h**) Evaluation of acetylated p53 by sandwich ELISA in mouse aortic SMCs. Left: western blot analysis of total p53 in the input cell lysates. Right: levels of acetylated p53 were measured by spectrophotometric determination of absorbance at 450 nm, normalized to input and expressed as fold change of control cells. (**i**) Acetylated p53 was evaluated as in **h**, in mouse aortic SMCs treated with vehicle (DMSO) or 10 μM RO5963, an Mdm2/Mdm4 inhibitor. In **h**,**i**, *P<0.05; NS, not significant, comparisons done by two-tailed *t*-test. In **b**,**c**,**f**, comparisons done by one-way ANOVA and Tukey's multiple comparisons test. In **b**–**d**, **f**–**i**, data represent the mean±s.e.m., and *n*=3.

*Cdkn1a (p21)* repression and *Ccnd1 (cyclin D1)* induction have been associated with activation of β-catenin/TCF signalling and proliferation in cultured SMCs^17^,^18^, so we tested expression of these genes, as well as that of proapoptotic *Bax* and antiapoptotic *Bcl2l1*, which have been related to β-catenin^23^,^24^. β-Catenin-deficient SMCs expressed higher mRNA levels of *Cdkn1a*, *Ccnd1*, *Bax*, and *Bcl2l1*, but lower levels of *Axin2*, a known target of β-catenin (Fig. 4a). Since increased *Ccnd1* and *Bcl2l1* levels could not explain the observed phenotype, we focused on *Cdkn1a* and *Bax* (direct targets of p53), and hypothesized that β-catenin might restrain p53 function to promote SMC survival and proliferation. Using a p53 reporter assay^25^, we found a substantially higher p53 transcriptional activity in β-catenin-deficient SMCs (Fig. 4b; Supplementary Fig. 4a)—importantly, *WT* SMCs treated with the same Ad-Cre protocol did not exhibit increased p53 activity (Supplementary Fig. 4b). Restoration of β-catenin function in β-catenin-deficient SMCs with β-catenin^S33Y^, a constitutively active form^26^, reduced p53 activity toward control levels (Fig. 4b); β-catenin^S33Y^ also abrogated p53 activity induced by p53 overexpression (Fig. 4c). In addition, SMCs treated with the β-catenin inhibitor XAV939 (ref. 27), which reduced β-catenin/TCF transcriptional activity in SMCs (Supplementary Fig. 4c), showed an increase in p53 activity (Fig. 4d). These observations indicate that β-catenin inhibits p53 activity in vascular SMCs.

P53 activation involves three steps, (1) stress-induced p53 accumulation by several mechanisms, many of which affect the ability of Mdm2 to ubiquitinate p53, (2) release of p53 from the Mdm2- and Mdm4-mediated repression, and (3) recruitment and interaction with numerous cofactors^4^. Surprisingly, β-catenin loss in SMCs neither increased p53 expression (Fig. 4e; Supplementary Fig. 4d), nor decreased the levels of Mdm2 and Mdm4 (Fig. 4e). Moreover, RO-5963—an Mdm2 and Mdm4 inhibitor^28^, at a dose sufficient to abrogate exogenous Mdm2 function in SMCs (Supplementary Fig. 4e)—did not increase p53 function in SMCs expressing physiologic levels of these ubiquitin ligases (Fig. 4f). Similarly, siRNA-mediated knockdown of Mdm2 and Mdm4 in SMCs failed to induce p53 activity (Supplementary Fig. 4f,g), which suggests that cultured SMCs do not require Mdm2/Mdm4 to restrain p53 activity.

These observations support the idea that β-catenin inhibits SMC p53 activity by a mechanism independent of effects on Mdm2 and Mdm4. Conceivably, this mechanism could limit p53 binding to DNA or decrease its recruitment of transcriptional coactivators. To separate these functions, we used a Gal4-binding domain-p53 fusion protein (Gal4BD-p53), and found that activity of this protein on a promoter driven via Gal4 response elements (pFR-Luc) was greater in β-catenin-deficient SMCs (Fig. 4g). Thus, β-catenin decreases p53 transcriptional activity through a post-translational mechanism that may affect recruitment of transcriptional coactivators. Acetylation of p53 is indispensable for its transcriptional activity, triggering recruitment of coactivators, among other functions^29^,^30^,^31^, so we assessed this modification in SMCs and found that β-catenin loss, but not Mdm2/Mdm4 inhibition, robustly increased p53 acetylation (Fig. 4h,i). Altogether, our findings support the idea that β-catenin indirectly inhibits p53 transcriptional activity in vascular SMCs not by limiting its abundance or potentiating Mdm2-/Mdm4-mediated repression, but through preventing p53 acetylation.

### β-catenin restrains p53 in SMCs during artery formation

**Figure 5:**
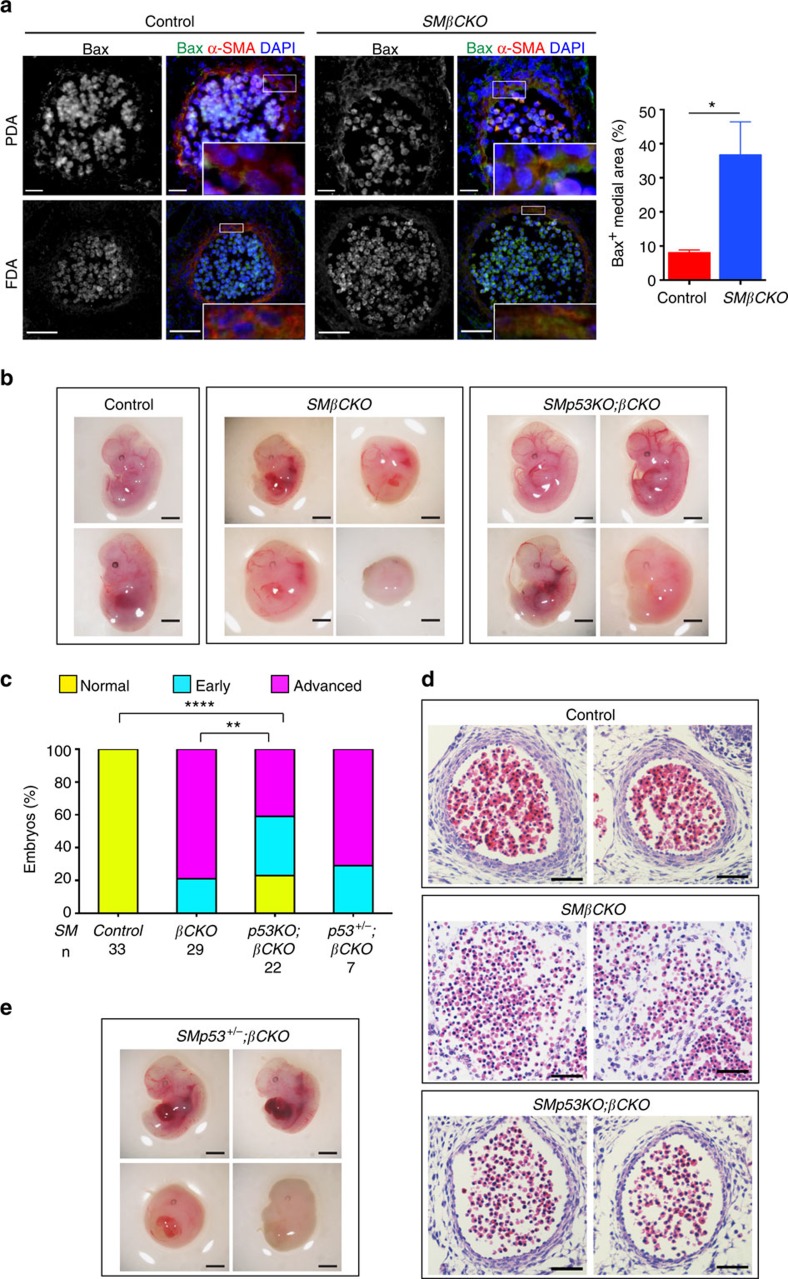
Loss of p53 suppresses the vascular phenotype caused by loss of β-catenin. (**a**) Left: immunostaining for Bax, a known p53 target gene, and α-SMA at E11.5. Scale bar, 20 μm (PDA); 50 μm (FDA). Right: quantification of Bax expression in the media of aortas, expressed as percentage of medial area positive for Bax. Data represent the mean ±s.e.m. *P<0.05 by two-tailed *t*-test. *n*=4. (**b**,**e**) E12.5 embryos of the indicated genotypes. Scale bar, 1 mm. (**c**) Percentage of E12.5 embryos of the indicated genotypes in three phenotypic categories: normal, early resorption or advanced resorption. **P<0.01; ****P<0.0001; comparisons done by *χ*^2^-test. (**d**) H&E-stained sections of FDAs of the indicated genotypes. Scale bar, 50 μm.

To evaluate p53 activity *in vivo*, we tested SMC expression of a known p53 target gene, *Bax*, in E11.5 embryos, and found that *SMβCKO* embryos exhibited higher expression than controls (Fig. 5a), consistent with increased p53 activity on loss of β-catenin. Thus, we hypothesized that repression of p53 is an essential aspect of the requirement for β-catenin in SMCs during artery formation. To test this idea, we studied mice with SM-selective inactivation of *Trp53* (*SMp53KO* mice), and with simultaneous inactivation of *Trp53* and *Ctnnb1* (*SMp53KO;βCKO*). *SMp53KO* mice developed normally (not shown), indicating that SMC p53 activity is not required for artery formation. To test if unrestrained p53 activity contributes to the phenotype seen with loss of SMC β-catenin, we compared *SMβCKO* and *SMp53KO;βCKO* embryos at E12.5, and noted differences in the degree of resorption. Among *SMβCKO* embryos, 21% were in an early stage of resorption, while 79% appeared advanced (Fig. 5b,c). Interestingly, development of *SMp53KO;βCKO* embryos was less affected: 23% looked normal, 36% were in early resorption and only 41% in advanced resorption—a clear difference from findings with *SMβCKO* embryos (Fig. 5b,c). Moreover, in *SMp53KO;βCKO* embryos, we observed aortas with multilayered walls, an observation not seen in any *SMβCKO* embryos at this stage (Fig. 5d), and with reduced Bax expression (Supplementary Fig. 5a–c). Thus loss of SMC p53 itself has no repercussion on vascular development, but suppresses the vascular phenotype resulting from inactivation of SMC β-catenin, supporting the idea that β-catenin represses p53 activity in SMCs *in vivo* during artery formation.

In addition, *SMp53^+/−^;βCKO* embryos phenocopied the *SMβCKO* embryos (Fig. 5c,e), indicating that a single *Trp53* allele provides sufficient p53 activity to prevent artery formation on loss of SMC β-catenin, and suggesting that low levels of p53 are effective in SMCs. Although the improvement in artery formation and overall development was important, p53 loss did not fully overcome the phenotype of *SMβCKO* embryos—thus, the critical requirement for SMC β-catenin during development extends beyond its suppression of p53 activity.

### SMC β-catenin C-terminal interactions inhibit p53 activity

**Figure 6:**
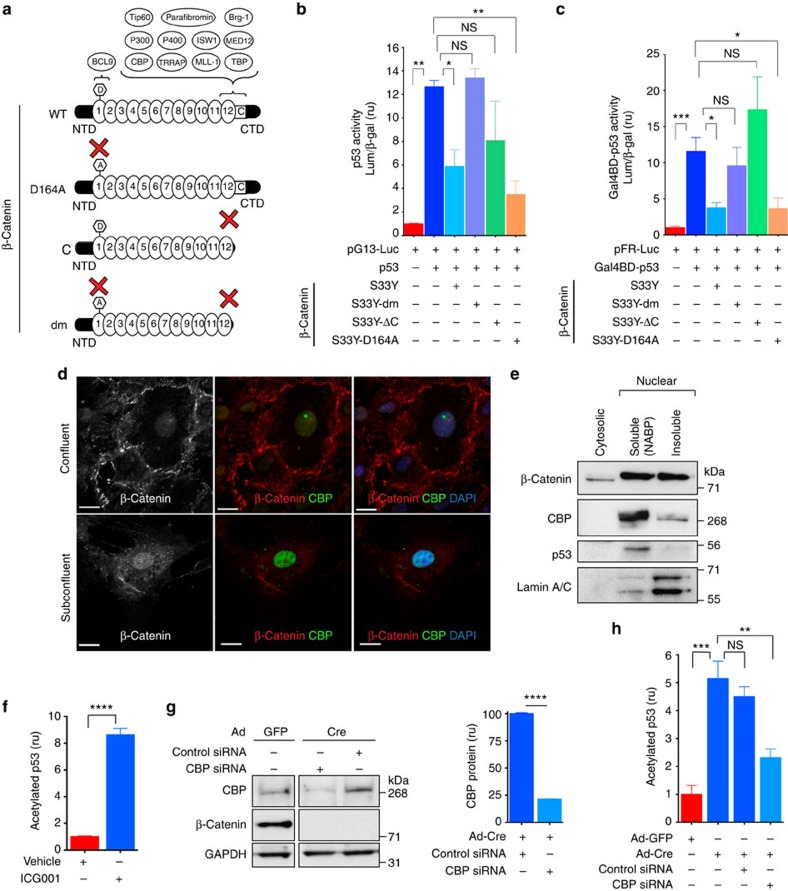
The β-catenin C-terminal interactions are required for repression of p53 activity. (**a**) Schematic of wild-type (WT) and mutant β-catenin proteins. (**b**,**c**) p53 activity and Gal4BD-p53 activity, respectively, in WT mouse aortic SMCs electroporated with the indicated expression vectors. *P<0.05; **P<0.01; ***P<0.001; comparisons done by one-way ANOVA and Dunnett's multiple comparisons test. In **b**, *n*=3; in **c**, *n*=6. Luminescence (Lum) was normalized to β-galactosidase activity (β-gal) to control for transfection efficiency. (**d**) Immunostaining of mouse aortic SMCs in confluent and subconfluent monolayers for β-catenin and CBP. Nuclear staining by DAPI. Scale bar, 20 μm. (**e**) Western blot analysis of indicated proteins in cytosolic and nuclear fractions of WT mouse aortic SMCs. (**f**) Evaluation of acetylated p53 by sandwich ELISA in WT mouse aortic SMCs treated with vehicle (DMSO) or 50 μM ICG001, an inhibitor of β-catenin/CBP interaction. Levels of acetylated p53 were measured by spectrophotometric determination of absorbance at 450 nm, normalized to input and expressed as fold change of control cells. ****P<0.0001 by two-tailed *t*-test. *n*=3. (**g**) Left: western blot analysis of indicated proteins in mouse aortic SMCs with annotated treatments. Right: densitometric analysis of normalized CBP protein levels in the indicated conditions. ****P<0.0001 by two-tailed *t*-test. *n*=3. (**h**) Evaluation of acetylated p53 as in **f**, in mouse aortic SMCs with indicated treatments. **P<0.01; ***P<0.001; comparisons done by one-way ANOVA and Dunnett's multiple comparisons test. *n*=3. In **b**,**c**,**f**–**h**, data represent the mean ±s.e.m. A, alanine residue in position 164; C, conserved helix C; CTD, carboxy-terminal domain; D, aspartate residue at position 164 on the first armadillo repeat; NABP, nucleic-acid-binding protein subset (soluble nuclear fraction); NS, not significant; NTD, amino-terminal domain. In **a** numbered ovals are armadillo repeats, and critical regions and respective interacting proteins are indicated by brackets.

Since β-catenin has two major functions in the cell—as a structural component in cadherin-mediated cell adhesion, and as a signalling factor acting as a transcriptional coactivator in the canonical Wnt pathway^10^—we wanted to know which function was required for p53 regulation. We recently developed and validated β-catenin mutants that partially or totally lack transcriptional activity, but have intact cell-adhesion function^32^: (1) *Ctnnb1*^*D164A*^, which has a single amino acid change (D164A) in the first armadillo repeat that prevents binding to the *N*-terminal coactivator B-cell lymphoma 9/BCL9L, and exhibits decreased transcriptional activity; (2) *Ctnnb1*^*ΔC*^, a C-terminal truncation that changes codon 673 to a premature stop signal, abrogating interaction with several C-terminal interacting coactivators, and shows decreased transcriptional activity; and (3) *Ctnnb1*^*dm*^, a double-mutant with both the D164A mutation and the C-terminal truncation, which lacks any detectable transcription-inducing activity (Fig. 6a). As above, we studied these signalling-specific mutations in vascular SMCs in the context of constitutively active β-catenin^S33Y^.

β-Catenin^dm^ did not repress p53 transcriptional activity (Fig. 6b), suggesting that β-catenin signalling function is essential, while its cell-adhesion role is not sufficient to restrain p53 activity. Signalling through β-catenin depends on protein–protein interactions with coactivators recruited by its N-terminal and C-terminal domains^10^,^33^. We used β-catenin^D164A^ and β-catenin^ΔC^ to compare the importance of N- or C-terminal interactions for p53 regulation. Similar to β-catenin^dm^, β-catenin^ΔC^ failed to reduce p53 activity, whereas β-catenin^D164A^ abrogated it (Fig. 6b). Moreover, while Gal4BD-p53 transcriptional activity was not affected by β-catenin^dm^ or β-catenin^ΔC^, a decrease was observed with β-catenin^D164A^ (Fig. 6c). These observations suggest that β-catenin C-terminal interactions are required to repress p53 activity in vascular SMCs, whereas N-terminal interactions are dispensable.

Moreover, the fact that β-catenin^dm^—which has an intact TCF-binding domain^10^,^32^—did not repress p53 activity also suggests that β-catenin/TCF interaction is not sufficient to fulfil this repressive function. To test this idea further, we used an inhibitor of the β-catenin/TCF complex, PKF118-310 (ref. 34). A dose of PKF118-310 that reduced β-catenin/TCF transcriptional activity in SMCs (Supplementary Fig. 6a) did not increase p53 activity in these cells expressing endogenous or induced levels of p53 (Supplementary Fig. 6b). Similarly, PKF118-310 decreased, rather than increased, Gal4BD-p53 activity (Supplementary Fig. 6c), suggesting that β-catenin/TCF interaction and associated Wnt target genes are not acting to restrain p53 activity in vascular SMCs.

Altogether these observations support the idea that β-catenin C-terminal interactions are necessary to restrain p53 activity through a β-catenin/TCF-independent mechanism. Interestingly, one of the known C-terminal interactors is the histone acetyltransferase Creb-binding protein (CBP)^10^,^35^, which also acetylates p53 (refs 29, 36). β-Catenin prevents p53 acetylation (Fig. 4h), so it is possible that β-catenin/CBP interaction limits CBP availability for interaction with and acetylation of p53 in the nucleus of vascular SMCs. Consistent with this idea, we found that both β-catenin and CBP localized to the nucleus of SMCs by immunofluorescence (Fig. 6d) and were present in the nuclear fraction of SMCs (Fig. 6e), and nuclear β-catenin expression was also observed in the wall of aortas of E12.5 control embryos (Supplementary Fig. 6d–f). Notably, blocking β-catenin/CBP interaction with ICG001 (ref. 37), which inhibited β-catenin/TCF transcriptional activity in SMCs (Supplementary Fig. 6g), increased p53 acetylation (Fig. 6f); moreover, knocking down CBP in β-catenin-deficient SMCs decreased acetylation of p53 (Fig. 6g,h)—while CBP knockdown in control SMCs (Ad-GFP) did not have any effect on their relatively low levels of acetylated p53 (Supplementary Fig. 6h).

### β-Catenin C-terminal interactions promote artery formation

To test the physiologic relevance of the above findings, we used mice in which mutant *Ctnnb1* alleles^32^, like those described above, provided the only source of β-catenin in SMCs. We crossed *Ctnnb1*^*dm/flox*^ and *Tagln-Cre;Ctnnb1*^*flox/WT*^ mice to produce *SMβC*^*dm*^ (SMC β-catenin replaced with the signalling-defective mutant), *SMβCKO* (complete loss of SMC β-catenin) and *Ctnnb1*^*flox/WT*^ (wild-type β-catenin) littermates. Interestingly, we found no *SMβC*^*dm*^ mice in postnatal screening (Supplementary Table 3, Supplementary Fig. 7a), indicating that these mutants were embryonic lethal.

**Figure 7:**
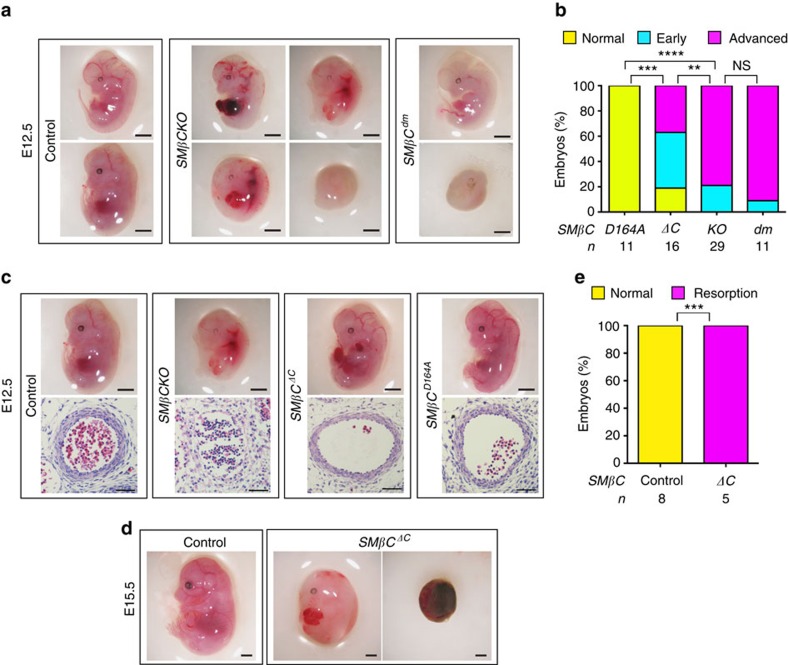
β-Catenin C-terminal signals in SMCs are essential for artery formation. (**a**) Mouse embryos of indicated genotypes, scale bar, 1 mm. (**b**) Percentage of E12.5 embryos of indicated genotypes in three phenotypic categories: normal, early resorption or advanced resorption. (**c**) Embryos of indicated genotypes and their FDA stained with H&E. Scale bar, 1 mm (embryos); 50 μm (arteries). (**d**) E15.5 embryos of indicated genotypes. Scale bar, 1 mm. (**e**) Percentage of E15.5 embryos of indicated genotypes in two categories: normal or resorption. Total embryos screened=29. In **b**,**e**: **P<0.01; ***P<0.001; ****P<0.0001; comparisons done by *χ*^2^-test or Fisher's exact test. NS, not significant.

Notably, all E12.5 *SMβC*^*dm*^ embryos were undergoing resorption—with 9% early and 91% advanced in their state of resorption. This finding was like that seen with *SMβCKO* embryos (Fig. 7a,b), and indicates that β-catenin's cell-adhesion function in SMCs is insufficient, whereas its signalling function is essential, for artery formation and normal embryogenesis.

To evaluate the importance of N- and C-terminal SMC β-catenin interactions *in vivo*, we designed breeding strategies to produce *SMβC*^*D164A*^ and *SMβC*^*ΔC*^ mice. In postnatal screening, these strategies yielded no *SMβC*^*ΔC*^ mice (Supplementary Table 4, Supplementary Fig. 7a), but normal appearing *SMβC*^*D164A*^ mice (Supplementary Table 5, Supplementary Fig. 7a) that reached adulthood. At E12.5, 19% of *SMβC*^*ΔC*^ embryos looked normal, 44% were in early resorption and 37% in advanced resorption—a profile somewhat milder than that of *SMβCKO* embryos (Fig. 7b). In contrast, all *SMβC*^*D164A*^ embryos appeared normal (Fig. 7b). Moreover, we found correlation between whole embryo phenotype and the structure of the FDA at E12.5: control embryos had a clear multilayered vessel wall, whereas *SMβCKO* aortas lacked SMC investment, as described before. In some *SMβC*^*ΔC*^ embryos, we observed an important, although incomplete, recovery of the vessel wall, while in the *SMβC*^*D164A*^ embryos, the aortic wall was fully restored (Fig. 7c). Since *SMβC*^*ΔC*^ embryos were embryonic lethal, but showed an intermediate phenotype at E12.5, we tested the idea that SMβC^ΔC^ could delay demise. At E15.5, a stage at which *SMβCKO* embryos were never observed, we found some *SMβC*^*ΔC*^ embryos, although all of them were undergoing resorption (Fig. 7d,e). These findings indicate that expression of the *Ctnnb1*^*ΔC*^ allele as the only source of β-catenin in SMCs ameliorates defective artery formation and delays embryonic death. On the other hand, *Ctnnb1*^*D164A*^ was sufficient for artery formation that supported embryonic and postnatal development in a proportion of mice; however, that proportion of *SMβC*^*D164A*^ mice observed in postnatal screening was lower than expected (Supplementary Fig. 7b). At E15.5, stage at which all *SMβC*^*ΔC*^ embryos were abnormal, all *SMβC*^*D164A*^ embryos looked normal (Supplementary Fig. 7c), suggesting that demise of some *SMβC*^*D164A*^ mice may occur perinatally or postnatally. Altogether, these *in vivo* studies support the idea that β-catenin signalling function in SMCs is essential for artery formation. Moreover, β-catenin C-terminal interactions are required to fulfil this signalling role; by comparison, β-catenin N-terminal interactions are not essential for early events of vascular maturation, but may contribute to survival at later stages of development.

## Discussion

This study demonstrates for the first time that SMC β-catenin is essential for developmental vascular maturation. SMC β-catenin deletion impairs artery formation with critical effects on SMC proliferation and survival. While other signalling pathways, including Notch^38^,^39^, sphingosine-1-phosphate^40^,^41^, PDGFB/PDGFRβ^42^,^43^,^44^ and TGF-β^8^,^45^,^46^,^47^ participate in vascular maturation, the phenotype due to SMC β-catenin inactivation is distinct in terms of timing, vessels affected and lack of effect on cellular differentiation.

Mechanistically, we found that β-catenin inhibits p53 activity in SMCs, and loss of this function impaired artery formation. Interestingly, postnatal increase in p53 activity inhibits angiogenesis^48^. Thus, repression of p53 activity appears necessary for developmental vascular maturation and for postnatal angiogenesis; our findings point to this as a key function of SMC β-catenin during development.

This functional connection between β-catenin and p53 is surprising. To our knowledge, such an interaction has not been demonstrated in vascular biology; moreover, it contrasts with interactions described in cancer, wherein β-catenin induces p53 stability and activity through a tumour-suppressor ARF-dependent effect^49^, or p53 downregulates β-catenin by promoting its degradation^50^. While β-catenin-mediated repression of p53 may be specific to SMCs, relevance of this mechanism to other biological contexts has yet to be tested, and could be of particular interest for stem cell biology^51^.

How β-catenin suppresses p53 function in SMCs is not fully elucidated, but our findings offer important clues. With β-catenin deficiency, p53 activity was higher, but its expression was unchanged. Since p53 abundance is tightly regulated by several mechanisms, many of which affect the ability of Mdm2 to ubiquitinate p53 (ref. 4), this lack of change in p53 levels suggests that β-catenin does not impinge on this complex system. In addition, β-catenin loss did not decrease expression of Mdm2 or Mmd4; and pharmacologic and genetic inhibition of Mdm2/Mdm4 did not increase p53 activity. Moreover, while β-catenin loss increased p53 acetylation, inhibition of Mdm2/Mdm4 did not. Thus, the mechanism leading to increased p53 activity on β-catenin deletion in vascular SMCs differs from that characterized by p53 accumulation and inhibition of Mdm2/Mdm4 function^4^. Our observations suggest that vascular SMCs in culture do not require Mdm2/Mdm4 function to repress p53 activity, but they do require β-catenin. Consistent with this idea, adult SMC-selective inactivation of Mdm4 has no repercussions, whereas Mdm2 SMC-selective inactivation induces death of intestinal SMCs without evidence of a vascular phenotype^52^; however, the low level of inactivation in this study limits conclusions about the role of Mdm2 in SMC biology in the adult vasculature. Whether Mdm2 and Mdm4 are required in vascular SMCs *in vivo* in particular during development has yet to be fully tested.

Our studies with mutant *Ctnnb1* alleles and inhibitors in vascular SMCs suggest that β-catenin N-terminal interactions and β-catenin/TCF interaction are dispensable to repress p53 activity, while C-terminal interactions are required. In particular, β-catenin forms that can interact with CBP also prevent p53 acetylation, a modification required for p53 activation^30^. In addition to CBP, other proteins interact with the β-catenin C-terminus^10^; while our findings with the ICG001 inhibitor are consistent with disruption of a direct β-catenin–CBP interaction, it is possible that other C-terminal interactors are involved and that the linkage between β-catenin and CBP is indirect. These additional factors participate in chromatin remodelling or connect β-catenin with the transcription initiation or elongation machinery; they are not β-catenin specific, and interact with other coactivators and transcription factors including p53 (refs 53, 54). Our studies with the Gal4BD-p53 chimeric protein reveal that β-catenin C-terminal interactions are required to repress p53 activity, independent of possible effects on DNA binding. In this context, it may be that p53 activity depends on recruitment of acetyltransferases and other coactivators necessary for transcription, and that the presence of the β-catenin C-terminus affects availability of acetyltransferases/coactivators in the SMC nucleus; β-catenin loss may increase this pool of acetyltransferases/coactivators and thereby potentiate p53 acetylation and activity. Alternatively, mechanisms involving altered expression of β-catenin–CBP-dependent, but TCF-independent, target genes whose products impinge on p53 acetylation and activity are also possible.

Notably, we found that SMC β-catenin C-terminal interactions are physiologically relevant for artery formation. Indeed, C-terminal interactions are required, while N-terminal interactions dispensable for arterial assembly; this is consistent with our cell culture studies and with the idea that mammals have a less strict requirement for β-catenin N-terminal signalling compared with *Drosophila*^55^.

Whereas *Ctnnb1*^*D164A*^ as the only source of β-catenin protein in SMCs fully restored artery formation, p53 loss in *SMβCKO* embryos resulted in an important but partial recovery, suggesting that β-catenin C-terminal interactions go beyond modulation of p53 activity. Such p53-independent mechanisms might involve β-catenin/TCF interaction and activation of β-catenin target genes. Although cyclin D1 has been characterized as a direct transcriptional target of β-catenin in studies using promoter-reporter constructs^56^, the unexpected increase in cyclin D1 mRNA observed in β-catenin-deficient SMCs is consistent with an indirect linkage between β-catenin and cyclin D1 expression^57^, and may be due in part to the higher fraction of β-catenin-deficient cells in the G0/G1 phase of the cell cycle, during which SMC cyclin D1 levels are high^58^.

Our studies indicate the significance of SMC β-catenin signalling function *in vivo* during arterial wall formation; however, relevant upstream regulators of this pathway remain unknown. Wnt ligands, released in an autocrine or paracrine (from endothelial cells or other sources) manner, might signal to SMCs for β-catenin stabilization and nuclear translocation during vascular maturation. This hypothesis remains to be tested.

SMC growth plays a pathogenic role in vascular disease^59^; our studies suggest that inhibition of β-catenin C-terminal interactions might serve as a therapeutic strategy. In addition, our findings show that SMC β-catenin is essential for the formation of multilayered arterial walls, which may be relevant for therapeutic angiogenesis and tissue engineering^60^,^61^. Moreover, tumour vasculature forms by angiogenesis and arterio-venogenesis^62^; our findings raise the possibility that SMC β-catenin contributes to formation and maturation of tumour vasculature by promoting SMC investment.

In conclusion, we have demonstrated that SMC β-catenin is essential for artery formation, in part by acting as a repressor of p53 and thereby promoting cell proliferation and survival. This novel role of β-catenin requires its signalling function, in particular, its C-terminal interactions. In addition to elucidating a critical function for β-catenin in development, these findings have implications for therapeutic angiogenesis, tissue engineering, tumour vascularization and vascular disease.

## Methods

### Mice

*Tagln-Cre*^20^, *Ctnnb1*^*flox/flox*^^21^ and *Trp53*^*flox/flox*^ mice^63^ as well as *Ctnnb1*^*D164A/WT*^*, Ctnnb1*^*ΔC/WT*^ and *Ctnnb1*^*dm/WT*^ mice^32^ have been described. *Tagln-Cre* mice (STOCK Tg(Tagln-cre)1Her/J, (C57BL/6 × SJL)F2) and *Ctnnb1*^*flox/flox*^ (B6.129-*Ctnnb1*^*tm2Kem*^/KnwJ) mice were obtained from the Jackson Laboratory. *Trp53*^*flox/flox*^ mice in a mixed C57BL/6 × 129 × FVB background (provided by Dr Liang Zhu, Albert Einstein College of Medicine) were crossed with *Ctnnb1*^*flox/flox*^ mice for four generations before use in experimental breeding, and the *Ctnnb1*^*D164A/WT*^, *Ctnnb1*^*ΔC/WT*^ and *Ctnnb1*^*dm/WT*^ were kept on a C57BL/6 background. In general, test genotypes were compared with littermate controls. Male and female 8–16–week-old mice were used as breeders for timed mating. Embryos from E9.5 to E19.5 and 21-day-old pups, male and female, were studied. Noontime of the day of detecting vaginal plugs was designated as E0.5. Mice and embryos were genotyped using tail or yolk sac DNA, respectively, plus validated PCR protocols (see references for each mouse line). All animals were housed in pathogen-free conditions, the procedures followed the rules and regulations of the AAALAC and were approved by the Institutional Animal Care and Use Committee (IACUC) of Albert Einstein College of Medicine.

### Histopathological and morphometric analysis

Embryos from E9.5 to E12.5 were dissected under a Stemi 2000-C stereomicroscope (Zeiss), fixed overnight with 4% formaldehyde in phosphate buffer (Fisher Scientific, SF100-4), dehydrated through an ethanol gradient, treated with xylene and embedded longitudinally in paraffin. Cross-sections (5 μm) were stained with hematoxylin and eosin. Hematoxylin and eosin-stained cross-sections of hearts and arteries were photographed with a digital microscope (COOLSCOPE, Nikon). Morphometric analysis of PDAs and FDAs, heart and OT was performed using ImageJ software. Total cross-sectional and vessel lumen areas were measured, with the distance between the total area edge and lumen edge indicating wall thickness. The total area of future left and right ventricles were measured, as well as the area of compacted and trabeculated myocardium in each ventricle. The ratio of myocardium area to total area was also calculated. The length of OT and OT wall thickness were also measured, and the latter was expressed as a fraction of the whole OT cross-section.

### Immunohistochemistry

Tissue sections were deparaffinized and rehydrated, endogenous peroxidase activity neutralized and antigens retrieved by boiling in sodium citrate solution (Vector Labs H-3300). Sections were blocked in 2% bovine serum albumin (BSA)/10% normal serum (same species of secondary antibody) and avidin-blocking solution (Vector) in phosphate-buffered saline (PBS), incubated with primary antibody in 2% BSA/10% normal serum and biotin-blocking solution (Vector) in PBS, incubated with biotinylated secondary antibody 1:500 (Vector), incubated with ABC (Vector PK-6100) or ABC-AP reagent (Vector AK-5000), incubated with DAB/substrate/chromogen system (Dako K3467; brown) or AP substrate kit I (Vector SK-5100 reagent, red) and counterstained with hematoxylin (Vector H-3404). Specificity of staining was confirmed by omission of the primary antibody. Images were obtained using a digital microscope (COOLSCOPE, Nikon). Primary antibodies used included anti-β-catenin (Santa Cruz sc-7963, 1:250 dilution), anti-α-SMA (Sigma A2547, 1:200 dilution), anti-Sm22α (Abcam 14106, 1:500 dilution), anti-CD31 (Abcam 28364, 1:50 dilution) and anti-pHH3 (Cell Signaling 9701, 1:50 dilution).

### Immunofluorescence

Tissue sections were deparaffinized and rehydrated, and antigen retrieval performed by boiling in sodium citrate solution (Vector Labs H-3300). Tissues were blocked in 0.3% Triton X-100, 2% BSA and 5% normal serum (same species as secondary antibody) in PBS; incubated with primary antibody in blocking solution; incubated with fluorochrome-conjugated secondary antibodies (Molecular Probes) in blocking solution; and stained with DAPI during mounting (FLUORO-GEL II, with DAPI, Electron Microscopy Sciences #17985-50). Fluorescent signals were visualized with Axio Observer.Z1 microscope (Zeiss). Subsequent image processing was performed with ImageJ. Specificity of staining was confirmed by omission of the primary antibody. Primary antibodies used: anti-β-catenin (Santa Cruz sc-7963, 1:50 dilution), anti-α-SMA (Santa Cruz sc-32351, 1:200 dilution), anti-cleaved Caspase-3 (Cell Signaling 9661, 1:50 dilution), anti-pHH3 (Cell Signaling 9701, 1:50 dilution), anti-Ki67 (Abcam 15580, 1:100 dilution), anti-Sm22α (Abcam 14106, 1:50 dilution) and anti-Bax (Abcam 32503, 1:50 dilution).

### TUNEL assay

An *in situ* cell death detection kit with fluorescein readout (Roche) was used as per the manufacturer's instructions. Briefly, tissue sections were deparaffinized and rehydrated, permeabilized by microwave irradiation in 0.1 M Citrate buffer (pH 6), labelled with TUNEL reaction mixture (label solution and terminal transferase), and analysed by fluorescence microscopy (Axio Observer.Z1 microscope, Zeiss). Sections incubated with label solution without transferase were used as negative controls. Sections incubated with DNase I before labelling procedures served as positive controls.

### Mouse aortic SMCs

Primary SMCs were obtained from 5 to 6-week-old *Ctnnb1*^*flox/flox*^ and *Ctnnb1*^*WT/WT*^ mouse aortas using collagenase-elastase digestion^64^ as follows: aortas were collected from the aortic arch to the iliac bifurcation, rinsed with cold serum-free DMEM with antibiotics, moved to the cell culture hood, stripped of adventitia, minced with scissors and digested with Collagenase 0.5 mg ml^−1^ (Sigma type I, C-0130) and Elastase 0.125  mg ml^−1^ (Sigma type III, E-0127) in serum-free DMEM with antibiotics at 37 °C until most cells were in suspension; then, enzymes were inactivated by adding DMEM with serum, and the cell suspension was recovered and centrifuged at 400*g* for 5 min, the SMC pellet was washed with complete medium, and SMCs plated in Fibronectin-coated dishes. Mouse aortic SMCs were maintained in DMEM (Invitrogen) containing 20% fetal bovine serum (FBS, HyClone), 100 U ml^−1^ penicillin, 100 μg ml^−1^ streptomycin and 1% L-glutamine, and subcultured weekly. Passages 2 or 3 were used for transduction with GFP- or Cre-expressing adenovirus (Ad5.CMV-GFP or Ad5.CMV-Cre) to obtain control or cells lacking β-catenin, respectively, and passages 4 to 8 were used for experiments. To evaluate cell population growth, 0.2 × 10^5^ cells of each group were plated in 35 mm dishes in triplicate for every time point, allowed to seed overnight, serum-starved for 48 h and then stimulated with and maintained in 20% FBS, and counted every 2 days using trypan blue exclusion and a hemocytometer. To evaluate cell population decline, 3 × 10^5^ cells of each group were plated in 35 mm dishes in triplicate for every time point, allowed to seed overnight, maintained under serum starvation and counted every day.

### Western blotting

Whole-cell protein lysates were extracted from SMCs using RIPA buffer (50 mM Tris-HCL pH 7.4, 1% NP40, 0.5% sodium deoxycholate, 0.1% SDS, 1 mM EDTA, 150 mM NaCl) with protease inhibitors (Complete, Roche). Protein concentrations were measured by a BCA protein assay kit (Pierce) and equal amounts of protein samples were loaded (20–60 μg), separated by 10% polyacrylamide gel electrophoresis and blotted onto PVDF membranes (Immobilon-P, Millipore). After blocking in TBST (Tris pH 8.0, NaCl 150 mM, 0.1% Tween 20) plus 4% (w/v) non-fat milk or BSA, blots were incubated at 4 °C overnight with primary antibodies. Signals were detected with horseradish peroxidase-conjugated secondary antibody and chemiluminescence (ECL, GE Healtcare). Equivalent protein loading was tested with anti-GAPDH (Santa Cruz, sc-25778, 1:5,000 dilution). Primary antibodies used: anti-β-catenin (Santa Cruz sc-7963, 1:500 dilution), anti-α-SMA (Sigma A2547, 1:5,000 dilution), anti-Sm22α (Abcam 14106, 1:10,000 dilution), anti-p53 (Santa Cruz sc-6243, 1:500 dilution), anti-MDM2 (Santa Cruz sc-965, 1:200 dilution), anti-MDMX (Santa Cruz sc-74468, 1:200 dilution), anti-Lamin A/C (Santa Cruz sc-6215, 1:200 dilution) and anti-CBP (Santa Cruz sc-369, 1:100 dilution). All uncropped blots can be found in Supplementary Fig. 8.

### Cell cycle analysis

SMCs (1 × 10^6^ cells) were incubated with 20 μM BrdU for 1 h, collected and fixed with ice-cold 70% ethanol while vortexing, then washed and incubated with 2N HCl/0.5% Triton X-100, resuspended in 0.1 M sodium tetraborate and resuspended in 0.5% Tween 20/1% BSA/PBS. In all, 20 μl of Anti-BrdU-FITC (Cat. No. 347583) were added and cells incubated for 30 min at room temperature, then cells were washed and resuspended in 0.5 ml of PBS containing 10 μg ml^−1^ RNase A and 10 μg ml^−1^ of propidium iodide for fluorescence-activated cell-sorting analysis in the Flow Cytometry Core Facility at Albert Einstein College of Medicine. Data were analysed with FlowJo 8.7 (FlowJo, LLC).

### Survival assay

SMCs (5 × 10^4^) were plated on a 22 × 22 mm coverslip in triplicate for each condition, allowed to become a subconfluent monolayer and then treated with 20 or 0% FBS for 48 h. Dead and live cells were distinguished by adding 150 μl of combined LIVE/DEAD assay reagents (2 μM Calcein AM and 4 μM ethidium homodimer-1) (L-3224, Life Techologies) to each coverslip. The percentage of dead cells for each coverslip was determined as the mean of (100 × dead/(live+dead)) of 16 randomly selected epifluorescent photomicrographic fields (Axio Observer.Z1 microscope, Zeiss). ImageJ software was used to count cells.

### Immunocytochemistry

SMCs were plated in chamber slides (BD Bioscience) 24 h before staining, washed with PBS and fixed with cold methanol, blocked with 3% normal goat serum/1% BSA/0.01% Triton X-100 in PBS, and incubated with anti-β-catenin (Santa Cruz sc-7963, 1:100 dilution), anti-CBP (Santa Cruz sc-369, 1:100 dilution) or anti-Sm22α (Abcam 14106, 1:200 dilution). Specific staining was identified with Alexa546 goat anti-mouse and Alexa488 goat anti-rabbit IgG (Molecular Probes). Samples were counterstained with DAPI and signals visualized with Axio Observer.Z1 microscope (Zeiss). Subsequent image processing was performed with ImageJ. Routine control experiments included omission of the primary antibodies.

### Transwell migration assay

A 6.5 mm Transwell system with 8.0 μm pore polyester membrane insert (Corning 3464) was used. SMCs were serum-starved for 24 h, collected and plated at 5 × 10^4^ cells per insert (upper chamber) in DMEM, as described before, containing either 0.2% or 20% FBS. The inserts were place into wells (lower chamber) containing either 0.2% or 20% FBS. After 8 h, cells remaining on top of the membrane were removed and cells that reached the bottom fixed and stained with Hema3 (Stat Pack Cat #123-869). The number of cells per high-power field ( × 200) was counted in 10 random fields per membrane. Triplicates for every genotype and condition were used every time.

### Luciferase assays

Vascular SMCs (5 × 10^4^ per well) were electroporated with pCMV-β-gal (transfection control) and with pG13-Luc (p53 activity-luciferase reporter, Addgene), pFR-Luc (Stratagene), M50 super 8 × TOPflash (β-catenin/TCF activity reporter, Addgene) or M51 Super 8 × FOPFlash (TOPflash mutant, Addgene). When indicated, pCMV-p53, pCMV-BD-p53, pcDNA3-β-catenin^S33Y^ (constitutively active β-catenin), pcDNA3-β-catenin^S33Y^-dm, pcDNA3-β-catenin^S33Y^-ΔC, pcDNA3-β-catenin^S33Y^-D164A, pCΦC-X2 (Mdm2-expressing vector) or empty vector were also included in the electroporation protocol. Vectors expressing wild-type and mutant β-catenin^S33Y^ have been previously validated^32^, as well as the Mdm2-expressing vector pCΦC-X2 (ref. 65, kindly provided by M. Oren). To generate the chimeric protein Gal4BD-p53, mouse p53 cDNA was generated by PCR from a mouse cDNA sample with primers containing *Hin*dIII and *Not*I sites to facilitate cloning (F 5′- gatcaagcttgactgccatggaggagtc -3′ and R 5′- gagatcgcggccgctagtcagtctgagt -3′), and the product was subcloned into the pCMV-BD vector (Stratagene). The electroporation was performed using the Neon transfection system (Invitrogen). Electroporated cells were plated in antibiotic-free medium in a 24-well plate for 24 h and then switched to regular medium. Triplicates for every group and condition were used every time. Cell lysates were collected 72 h after electroporation and luciferase activity was determined using the Glo-lysis buffer system (Promega) and the Synergy 2 Microplate Reader (BioTek). Luciferase activities were normalized to β-galactosidase activity for each well to control for transfection efficiency. When indicated, cells were treated as follows: (i) with validated doses of XAV939 (Cayman Chemical 13596; 1 or 10 μM), RO-5963 (Calbiochem 44153; 5, 10 or 20 μM), PKF118-310 (Sigma K4394; 0.7 μM) or vehicle; (ii) serum-starved 24 h before collecting; (iii) transfected with Mdm2/Mdm4 or both, or control Ambion Silencer Select Pre-designed siRNAs using X-tremGENE siRNA transfection reagent (Roche 04476093001).

### Real-time PCR

Total RNA was isolated from SMCs by homogenization in TRIzol (Invitrogen), treated with DNase I (1 U μl^−1^, Promega) and used for first-strand cDNA synthesis (SuperScriptIII, Invitrogen). Quantitative PCR was performed using a SYBR Green qPCR kit (Stratagene) and Mx3000P Real-Time PCR system (Stratagene). mRNA levels were normalized to *Rps13* and expressed as relative values in comparison with the specific control group using the comparative ΔΔC_t_ method (User Bulletin #2, ABI Prism 7700, Applied Biosystems). Primer sequences were as follows: *Axin2* (F 5′- gagagtgagcggcagagc -3′, R 5′- cggctgactcgttctcct -3′), *Bax* (F 5′- gtgagcggctgcttgtct -3′, R 5′- ggtcccgaagtaggagagga -3′), *Bcl2l1* (F 5′- tgaccacctagagccttgga -3′, R 5′- tgttcccgtagagatccacaa -3′), *Ccnd1* (F 5′- gagattgtgccatccatgc -3′, R 5′- ctcctcttcgcacttctgct -3′), *Cdkn1a* (F 5′- aacatctcagggccgaaa -3′, R 5′- tgcgcttggagtgatagaaa -3′), *Rps13* (F 5′- tgctcccacctaattggaaa -3′, R 5′- cttgtgcacacaacagcattt -3′) and *Trp53* (F 5′- acgcttctccgaagactgg -3′, R 5′- agggagctcgaggctgata -3′).

### Acetylation of p53

Evaluation of p53 acetylation in SMCs was done by a PathScan Acetylated p53 Sandwich ELISA kit (Cell Signaling 7236C), following manufacturer instructions. Briefly, total cell lysates were obtained, sonicated and protein concentration measured. Then, 100 μl of each sample containing equal amount of protein was added to a microwell coated with a p53 mouse monoclonal antibody and incubated at 4 °C overnight. The microwells were extensively washed, and an acetylated-lysine rabbit monoclonal antibody added for 1 h at 37 °C. After washing, an anti-rabbit horseradish peroxidase-linked antibody was added for 0.5 h at 37 °C. After washing, 100 μl of TMB substrate was added for 10 min at 37 °C. Then, 100 μl of STOP solution was added and absorbance at 450 nm read in a Synergy 2 Microplate Reader (BioTek). The magnitude of the absorbance is proportional to the quantity of acetylated p53. Triplicates for every genotype and condition were used every time. When indicated, cells were treated with 10 μM RO5963 (Calbiochem 44153), 50 μM of ICG001 (SelleckBio S2662) or vehicle (DMSO); or transfected with CBP siRNA (Santa Cruz sc-29243) or control siRNA (Santa Cruz sc-44231) using X-tremGENE siRNA transfection reagent (Roche 04476093001).

### Isolation of nuclear fraction

Control mouse aortic SMCs were collected by trypsinization, washed with ice-cold PBS and collected by gentle centrifugation. Isolation of cellular fractions was done using the Qproteome nuclear protein kit (QIAGEN 37582), following manufacturer's instructions. Briefly, 5 × 10^6^ SMCs were resuspended in hypotonic Lysis buffer NL. Then detergent was added and cells were vortexed to rupture the plasma membrane. Centrifugation was used to separate the cytosolic fraction (supernatant) from nuclei (pellet). The nuclear pellet was washed and incubated in a high-salt buffer. Centrifugation was used to separate the nucleic-acid-binding proteins (supernatant) from the nuclear debris (including genomic DNA). Extraction of the ‘insoluble' nuclear proteins was done by incubating nuclear debris with a buffer containing Benzonase, which digests DNA and releases proteins intimately associated with DNA.

### Statistics

Comparisons between two groups were evaluated by two-tailed *t*-test, and between more than two groups by one-way or two-way ANOVA (analysis of variance) followed by *post hoc* test when appropriate. Analysis of phenotypic categories and comparison of observed versus expected genotype frequencies was done by *χ*^2^-test or Fisher's exact test. Significance was accepted for values of *P*<0.05. All *in vitro* experiments were replicated at least three independent times. For developmental animal studies, no statistical method was used to predetermine sample size. In general, breeding strategies were designed to produce embryos with the genotypes of interest and controls within the same litter—thus littermates were compared—and no randomization was necessary to allocate animals in experimental groups. No embryos were excluded from the analysis, and investigators were not blinded.

### Data availability

The authors declare that all data supporting the findings of this study are available within the article and its Supplementary Information files or from the corresponding author on reasonable request.

## Additional information

**How to cite this article:** Riascos-Bernal, D.F. *et al*. β-Catenin C-terminal signals suppress p53 and are essential for artery formation. *Nat. Commun.* 7:12389 doi: 10.1038/ncomms12389 (2016).

## Supplementary Material

Supplementary Information Supplementary Figures 1-8, Supplementary Tables 1-5

## Figures and Tables

**Table 1:** Mouse genotype abbreviations.

**Genotype**	**Abbreviation**	**β-Catenin protein expressed in SMCs** ***in vivo***
*Tagln-Cre;Ctnnb1*^*flox/flox*^	*SMβCKO*	None
*Tagln-Cre;Ctnnb1*^*dm/flox*^	*SMβC*^*dm*^	Only β-catenin^dm^
*Tagln-Cre;Ctnnb1*^*D164A/flox*^	*SMβC*^*D164A*^	Only β-catenin^D164A^
*Tagln-Cre;Ctnnb1*^*ΔC/flox*^	*SMβC*^*ΔC*^	Only β-catenin^ΔC^
*Tagln-Cre;Trp53*^*flox/flox*^	*SMp53KO*	WT
*Tagln-Cre;Trp53*^*flox/flox*^*;Ctnnb1*^*flox/flox*^	*SMp53KO;βCKO*	None
*Tagln-Cre;Trp53*^*flox/WT*^*;Ctnnb1*^*flox/flox*^	*SMp53*^*+/−*^;*βCKO*	None
