# Supplementary material for: β-Catenin C-terminal signals suppress p53 and are essential for artery formation
Source: Nat Commun. 2016 Aug 8;7:12389. doi: 10.1038/ncomms12389 (PMC4979065; doi:10.1038/ncomms12389)
Supplement: Supplementary Information — Supplementary Figures 1-8, Supplementary Tables 1-5 [file ncomms12389-s1.pdf]

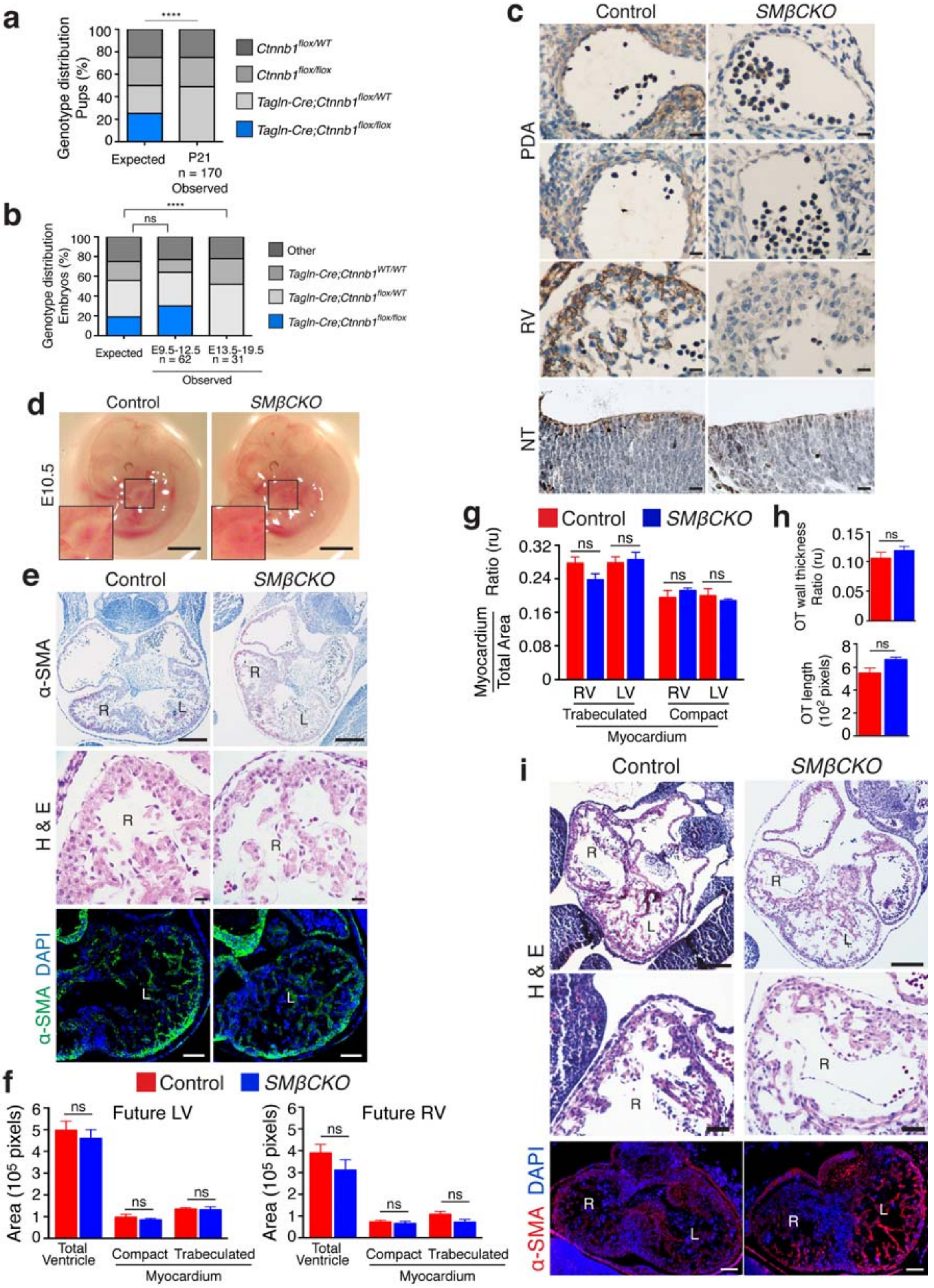

a
Genotype distribution Pups (%)
Expected
P21 n = 170 Observed
Ctnnb1flox/WT
Ctnnb1flox/flox
Tagln-Cre;Ctnnb1flox/WT
Tagln-Cre;Ctnnb1flox/flox
b
Genotype distribution Embryos (%)
Other
Tagln-Cre;Ctnnb1WT/WT
Tagln-Cre;Ctnnb1flox/WT
Tagln-Cre;Ctnnb1flox/flox
Expected
E9.5-12.5 n = 62
E13.5-19.5 n = 31
Observed
c
Control
SMβCKO
PDA
RV
NT
d
E10.5
e
α-SMA
H & E
α-SMA DAPI
f
Future LV
Future RV
Area (10^5 pixels)
Total Ventricle
Compact
Trabeculated
Myocardium
g
Myocardium/Total Area Ratio (ru)
Trabeculated
Compact
h
OT wall thickness Ratio (ru)
OT length (10^2 pixels)
i

Supplementary Figure 1

Loss of SMC β-catenin causes embryonic demise without a major cardiac phenotype. (**a**) Observed vs expected distribution of genotypes in 21 day-old (P21) pups born from crossing *Tagln-Cre;Ctnnb1*$^{flox/WT}$ with *Ctnnb1*$^{flox/flox}$ mice. (**b**) Observed vs expected distribution of genotypes in E9.5-E12.5 or E13.5-E19.5 embryos from crossing *Tagln-Cre;Ctnnb1*$^{flox/WT}$ with *Tagln-Cre;Ctnnb1*$^{flox/WT}$ mice. In **a** and **b**: ns, not significant; ****, p<0.0001 by Chi-square test. (**c**) Immunohistochemistry (IHC) for β-catenin (brown). PDA, paired dorsal aorta. RV, future right ventricle. NT, neural tube. Scale bar = 20 μm. (**d**) E10.5 embryos of indicated genotypes, which appear grossly normal, without signs of edema. Cardiac region (inset) does not show pericardial effusion. Scale bar = 1 mm. (**e**) Hearts at E10.5 of indicated genotypes stained for α-smooth muscle actin (α-SMA) by IHC (red) or immunofluorescence (green), or H&E, as annotated. Scale bars: Top = 200 μm, middle = 20 μm, and bottom = 100 μm. (**f**) Total area and areas of trabeculated and compact myocardium of future left ventricle (LV) and RV in E10.5 hearts. (**g**) Trabeculated or compact myocardium area to total ventricular area ratio in E10.5 hearts. In **f** and **g**: ns, not significant; two-way ANOVA with Sidak's multiple comparison test. (**h**) Top: Thickness of the outflow tract (OT) wall to total OT cross section ratio at E10.5. Bottom: Length of the OT at E10.5. ns, not significant; two-tailed t test. (**i**) Hearts at E11.5 of indicated genotypes stained with H&E or for α-SMA (red) and DAPI (blue) by immunofluorescence. Scale bars: Top = 200 μm, middle = 40 μm, bottom = 100 μm. In **e** and **i**: R, future right ventricle. L, future left

ventricle. In **f**, **g** and **h**: data represent the mean +/- s.e.m. n = 5 for control; n = 4 for *SMβCKO.*

Supplementary Figure 2

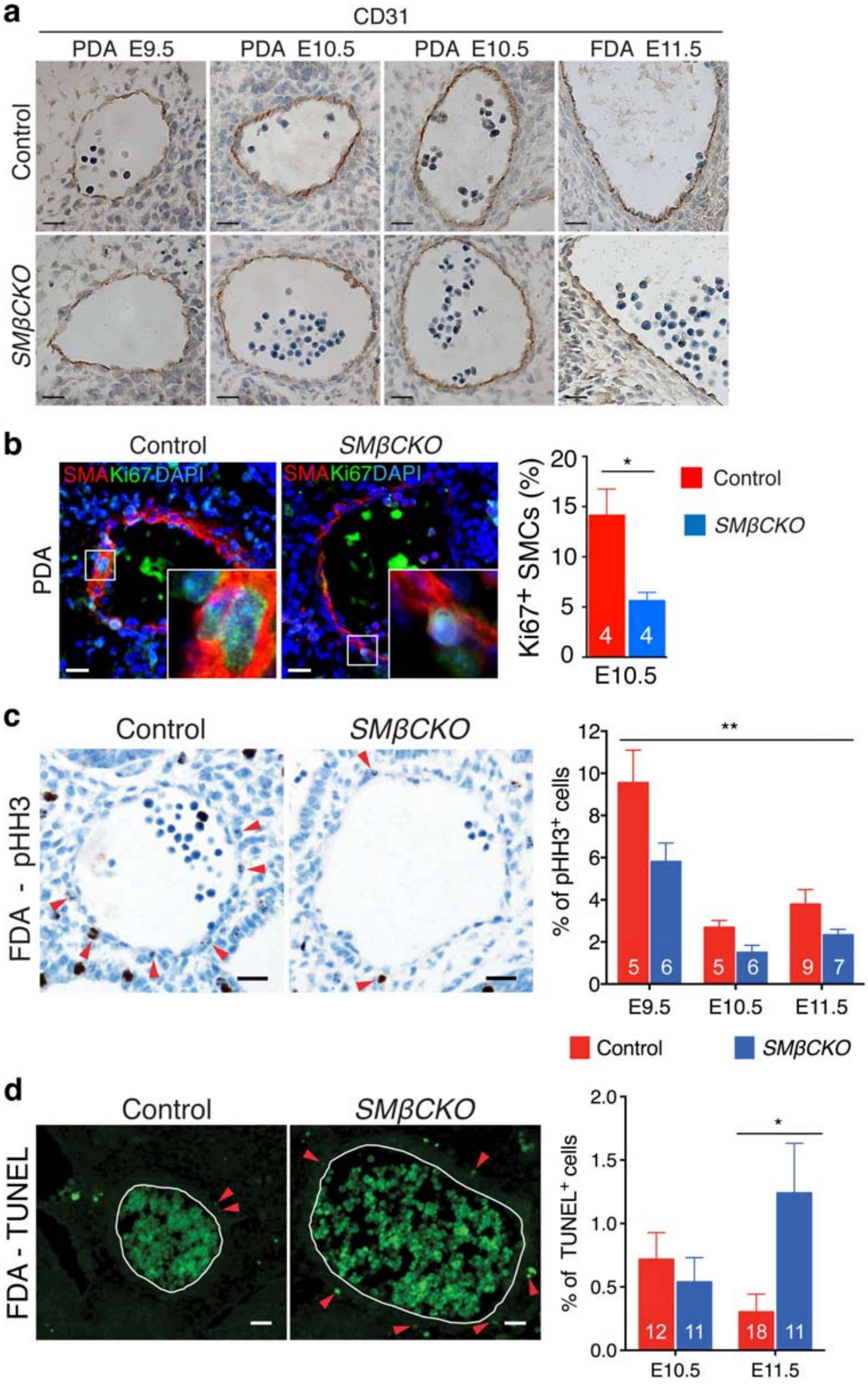

Supplementary Figure 2

Loss of SMC β-catenin impairs SMC cell proliferation and survival in the arterial wall.

(**a**) Immunohistochemistry (IHC) for the endothelial marker CD31 (brown). Scale bar = 20 μm. PDA, paired dorsal aorta. FDA, fused dorsal aorta. (**b**) Left: Immunostaining for Ki67, proliferation marker, and α-SMA, SMC marker, in PDAs at E10.5. Scale bar = 20 μm. Right: Quantification of Ki67$^+$ SMCs in PDAs. *, $p<0.05$ by two-tailed t test. (**c**) Left: IHC for the mitotic marker phospho-Histone H3 (pHH3) at E9.5. Arrowheads indicate pHH3$^+$ cells. Scale bar = 25 μm. Right: Quantification of pHH3$^+$ cells in the wall of the FDA. **, $p<0.01$ comparing genotypes by two-way ANOVA. (**d**) Left: TUNEL assay, arrowheads indicate TUNEL$^+$ cells. The white line delineates the lumen. Scale bar = 25 μm. Right: Quantification of TUNEL$^+$ cells in the wall of the FDA. *, $p<0.05$ by two-way ANOVA and Bonferroni's multiple comparisons test. In **b**, **c** and **d**, data represent the mean +/- s.e.m., the n numbers are indicated within the bars.

Supplementary Figure 3

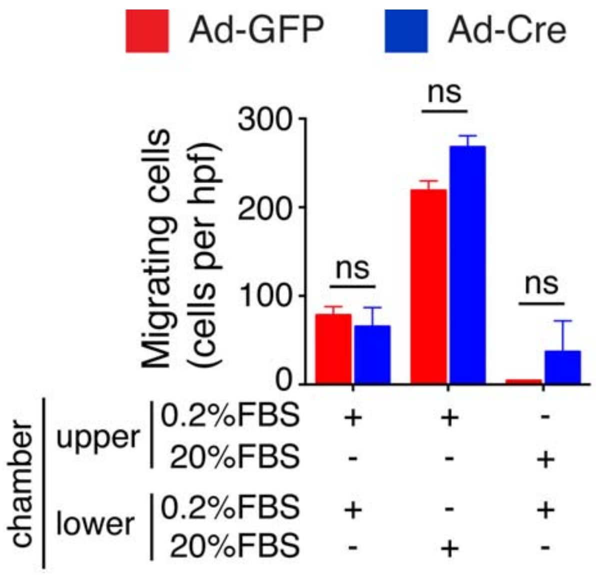

Supplementary Figure 3

Loss of β-catenin does not impair migration of vascular SMCs.

Transwell migration assay of mouse aortic control SMCs (Ad-GFP) and β-catenin-deficient SMCs (Ad-Cre) with the indicated conditions in the insert (upper chamber and well (lower chamber). FBS, fetal bovine serum; hpf, high power field (200X). ns, not significant. Comparisons done by two-way ANOVA and Sidak's multiple comparisons test. n = 4. Data represent the mean +/- s.e.m.

Supplementary Figure 4

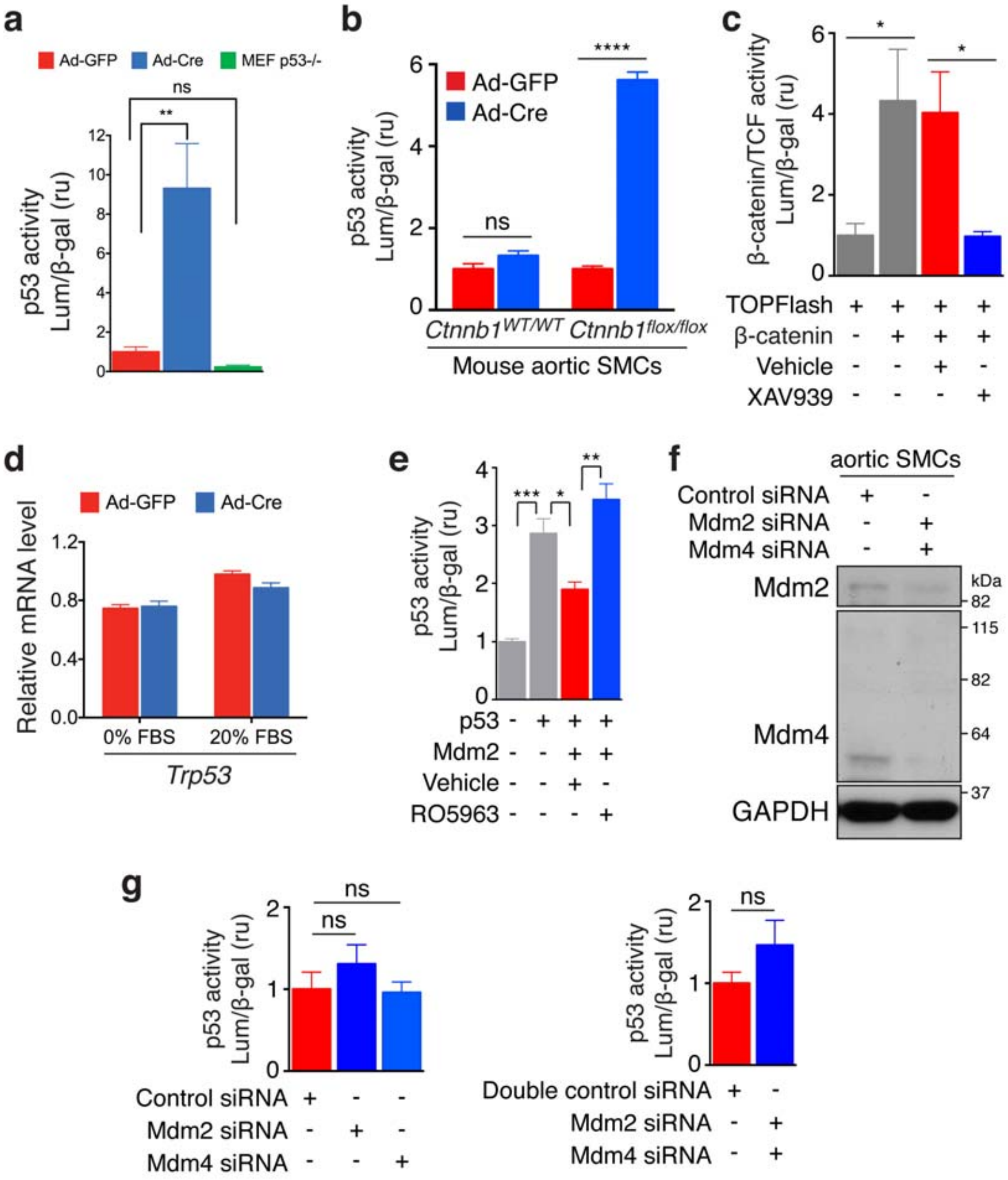

Supplementary Figure 4

β-catenin restrains p53 transcriptional activity in vascular SMCs.

(**a**) p53 transcriptional activity measured with the p53 reporter plasmid, PG13-Luc, in mouse aortic SMCs, Ad-GFP (control) vs Ad-Cre (β-catenin deficient), and p53 null

mouse embryonic fibroblasts (MEFs p53$^{-/-}$). **, p<0.01; ns, not significant by one-way ANOVA and Tukey's multiple comparisons test. n = 6. (**b**) p53 transcriptional activity measured as above in mouse aortic SMCs with indicated genotypes and treatments. ****, p<0.0001 by two-way ANOVA and Sidak's multiple comparisons test. n = 6 for *Ctnnb1*$^{WT/WT}$ SMCs and n = 3 for *Ctnnb1*$^{flox/flox}$ SMCs. (**c**) β-catenin/TCF transcriptional activity measured with the TOPflash reporter in mouse aortic SMCs electroporated with indicated expression vectors and treated with 10 µM XAV939 (β-catenin inhibitor) or vehicle (DMSO). *, p<0.05 by one-way ANOVA and Tukey's multiple comparisons test. n = 3. (**d**) RT-qPCR for *Trp53* in mouse aortic SMCs, Ad-GFP (control) vs Ad-Cre (β-catenin deficient), normalized to *Rps13*. Data represent the mean +/- s.d. n = 3. FBS, fetal bovine serum. (**e**) p53 transcriptional activity measured as before in control mouse aortic SMCs electroporated with indicated expression vectors and treated with vehicle (DMSO) or 10 µM RO5963 (Mdm2/Mdm4 inhibitor). *, p<0.05; **, p<0.01; ***, p<0.001 by one-way ANOVA and Tukey's multiple comparisons test. n = 3. (**f**) Western analysis of siRNA-mediated Mdm2/Mdm4 knockdown in mouse aortic SMCs. (**g**) p53 activity in mouse aortic SMCs with siRNA-mediated knockdown of Mdm2 or Mdm4 (left) or Mdm2 plus Mdm4 (right). ns, not significant by one-way ANOVA and Tukey's multiple comparisons test (left) or by two-tailed t test (right), n = 6. In **a-c**, **e** and **g**: The luminescence (Lum) signal was normalized to β-galactosidase activity (β-gal) to control for transfection efficiency, and data represent the mean +/- s.e.m.

Supplementary Figure 5

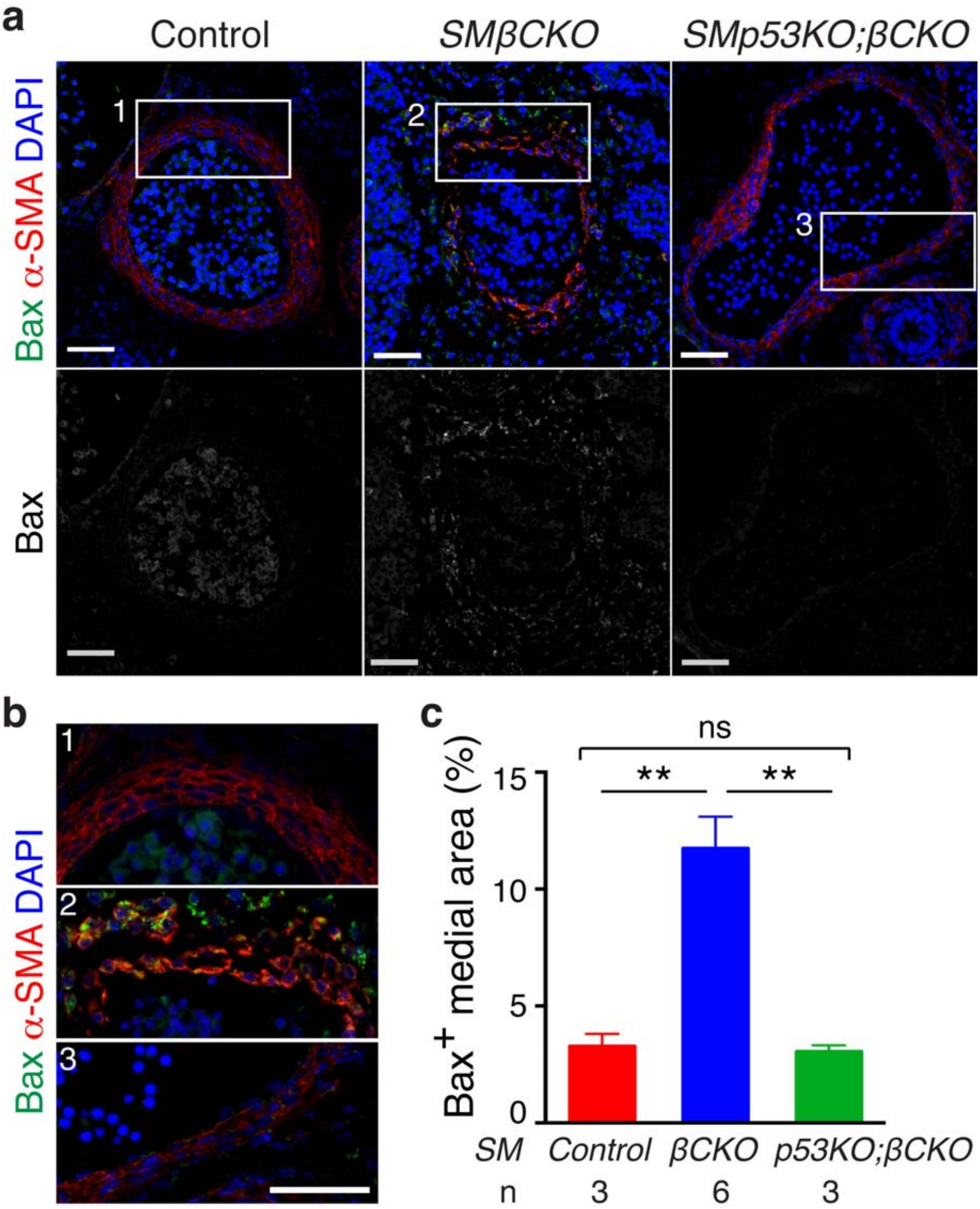

Supplementary Figure 5

Loss of p53 suppresses the increased Bax expression induced by loss of β-catenin.

(**a**) Immunostaining for Bax, a known p53 target gene, and α-SMA at E12.5 in fused dorsal aortas (FDA). Scale bar = 50 μm. (**b**) Enlarged regions indicated in **a**, scale bar = 50 μm. (**c**) Quantification of Bax expression in the media of aortas, expressed as % of medial area positive for Bax. ns, not significant; **, p<0.01 by one- way ANOVA and Tukey's multiple comparisons test. Data represent the mean +/- s.e.m.

Supplementary Figure 6

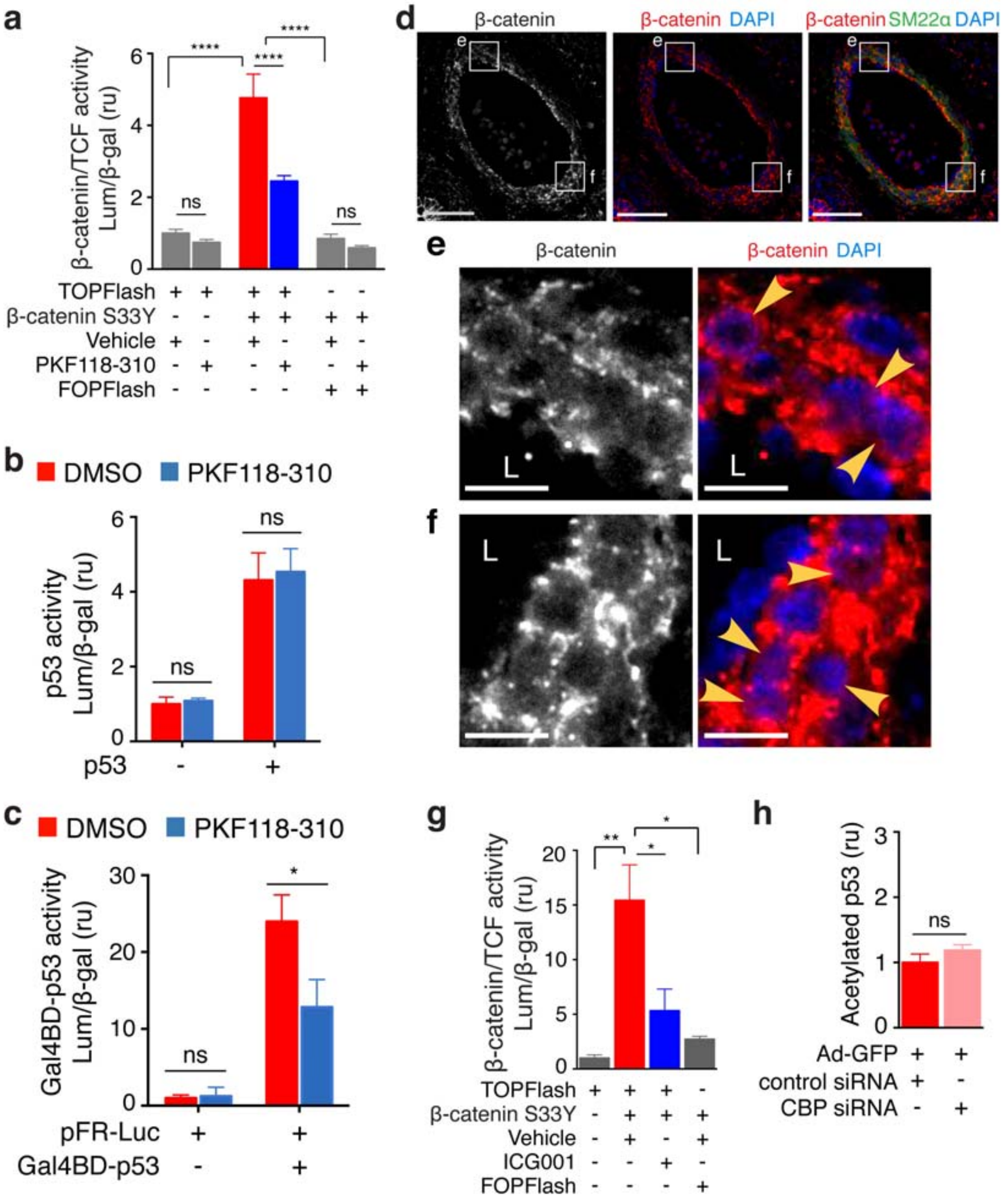

Supplementary Figure 6

β-catenin/TCF interaction is dispensable for inhibition of p53 activity in SMCs

(**a**) β-catenin/TCF transcriptional activity measured with the TOPflash reporter in mouse aortic SMCs electroporated with indicated expression vectors and treated

with 0.7 µM PKF118-310 (inhibitor of β-catenin/TCF complex) or vehicle (DMSO). The FOPFlash vector carries mutated TCF response elements unable to bind TCF and therefore serves as an additional control for specificity. ****, $p<0.0001$. n = 3. (**b**) p53 activity measured with the p53 reporter plasmid, PG13-Luc, in WT mouse aortic SMCs treated with vehicle (DMSO) or 0.7 µM PKF118-310 and electroporated with empty vector or p53-expressing vector. n = 3. (**c**) Gal4BD-p53 activity in WT mouse aortic SMCs electroporated with the indicated expression vectors, and treated with vehicle (DMSO) or 0.7 µM PKF118-310. n = 6. In **a**-**c**: Comparisons done by two-way ANOVA and Sidak's multiple comparisons test. (**d**) Immunostaining for β-catenin and Sm22α in dorsal aortas of control embryos. Scale bar = 50 µm. (**e** and **f**) Enlarged images of indicated regions in **d**. Arrowheads indicate SMCs with nuclear β-catenin. L, lumen. Scale bar = 10 µm. (**f**) β-catenin/TCF transcriptional activity measured with the TOPflash reporter in mouse aortic SMCs electroporated with indicated expression vectors and treated with 50 µM ICG001 (inhibitor of β-catenin/CBP interaction) or vehicle (DMSO). *, $p<0.05$; **, $p<0.01$ by one-way ANOVA and Tukey's multiple comparisons test. n = 3. (**g**) Evaluation of acetylated p53 by sandwich ELISA in control (Ad-GFP) mouse aortic SMCs with indicated treatments. Levels of acetylated p53 were measured by spectrophotometric determination of absorbance at 450nm, normalized to input, and expressed as fold change of control-treated cells. ns, not significant by two-tailed t test. n = 3. In **a**-**c** and **g**: Luminescence (Lum) was normalized to β-galactosidase activity (β-gal) to control for transfection efficiency. In **a**-**c**, **g** and **h**: data represent the mean +/- s.e.m.

Supplementary Figure 7

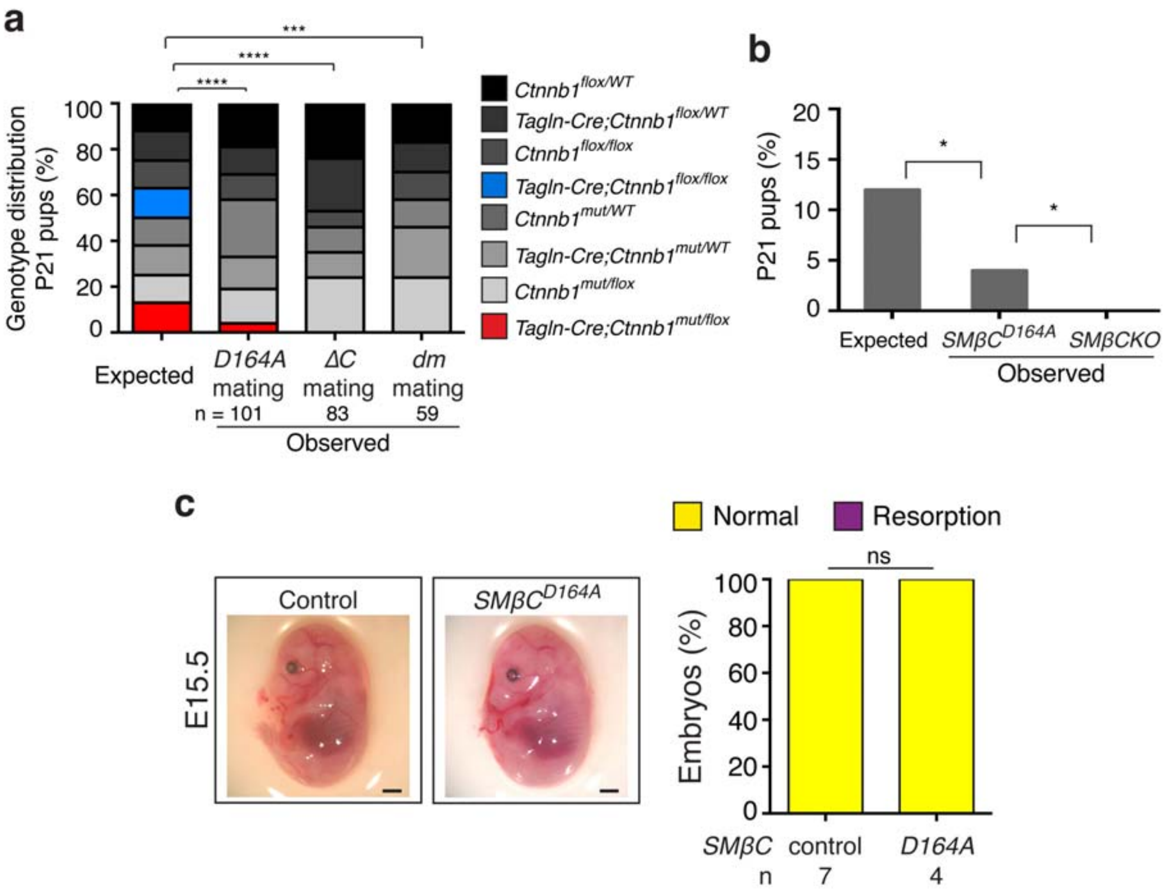

Supplementary Figure 7

SMβC^{D164A} mice appear normal at E15.5 and in a postnatal screen.

(**a**) Observed vs expected distribution of genotypes in 21 day-old (P21) pups born from crossing *Tagln-Cre;Ctnnb1*$^{flox/WT}$ with *Ctnnb1*$^{mut/flox}$ mice; where "*mut*" indicates either *D164A*, Δ*C* or *dm Ctnnb1* mutant alleles in three independent mating strategies. ***, p<0.001; ****, p<0.0001 by Chi-square test. (**b**) Observed vs expected frequency of P21 *SMβC*^{D164A} mutants and *SMβCKOs*. According to the mating strategy both genotypes have the same expected frequency, but only normal-appearing *SMβC*^{D164A} mutants were observed, although at a significant lower

frequency than expected. *, $p<0.05$ by Chi-square test. (**c**) Left: E15.5 embryos of indicated genotypes, scale bar = 1mm. Right: Percentage of E15.5 embryos of indicated genotypes in two categories: normal or resorption. Total embryos screened = 26. ns, not significant by Fisher's exact test.

Supplementary Figure 8

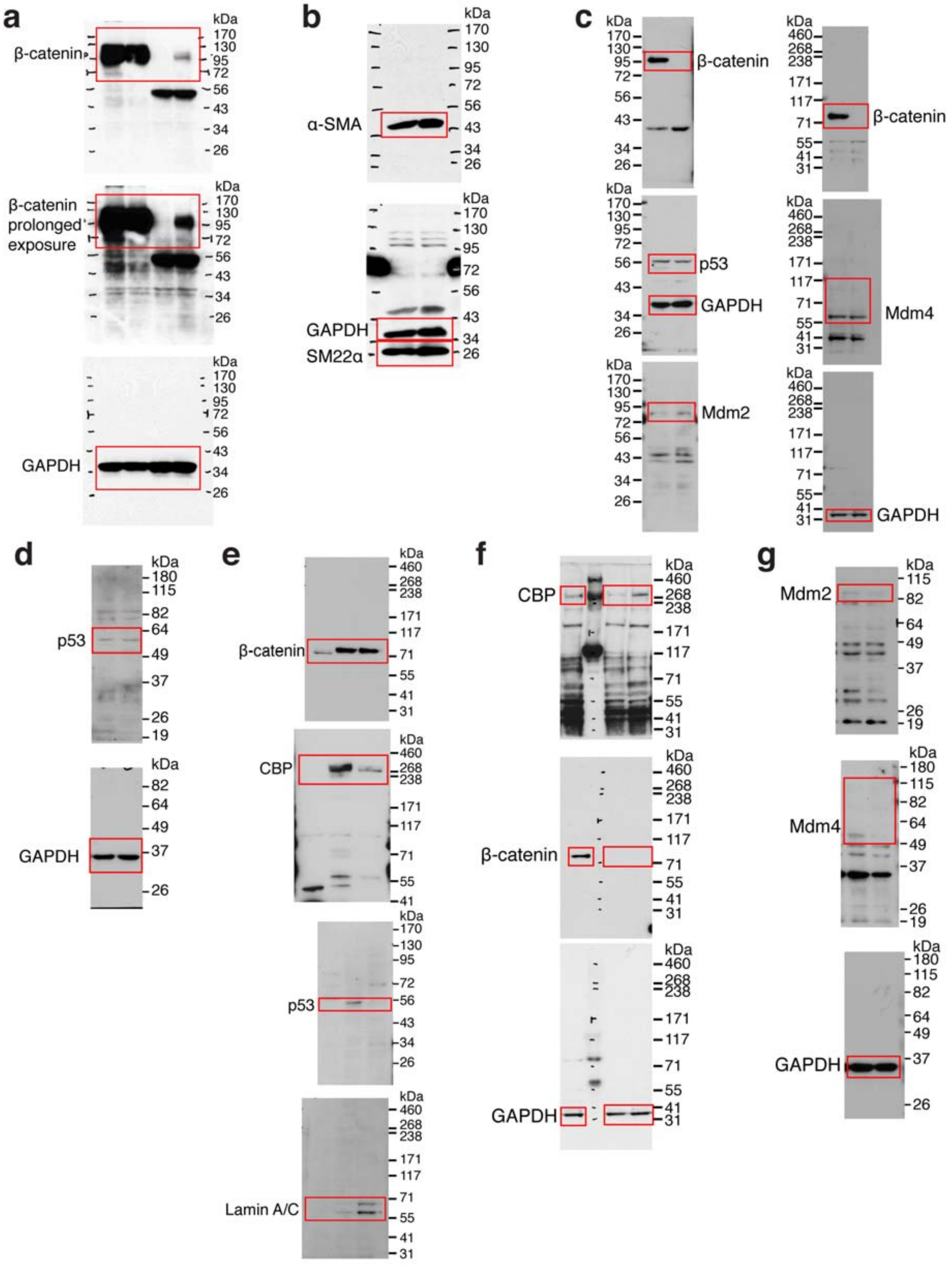

Supplementary Figure 8

Uncropped lanes of Western blots presented in the manuscript.

(**a**) Blots of Fig. 3a. (**b**) Blots of Fig. 3g. (**c**) Blots of Fig. 4e. (**d**) Blots of Fig. 4h. (**e**) Blots of Fig. 6e. (**f**) Blots of Fig. 6g. (**g**) Blots of Supplementary Fig. 4f. Red boxes indicate areas presented in the respective figures.

Supplementary Table 1

Observed genotype distribution of 21 day old mouse pups from different breeding strategies.

Possible Genotypes							
**Breeding strategy**	*Ctnnb1*$^{flox/flox}$	*Tagln-Cre; Ctnnb1*$^{flox/flox}$	*Ctnnb1*$^{flox/WT}$	*Tagln-Cre; Ctnnb1*$^{flox/WT}$	*Ctnnb1*$^{WT/WT}$	*Tagln-Cre; Ctnnb1*$^{WT/WT}$	**Subtotal**
Ctnnb1$^{flox/WT}$ X *Tagln-Cre; Ctnnb1*$^{flox/WT}$	2 (7%)	0	9 (30%)	4 (13%)	10 (33%)	5 (17%)	30
Tagln-Cre; Ctnnb1$^{flox/WT}$ X *Tagln-Cre; Ctnnb1*$^{flox/WT}$	1 (7%)	0	2 (13%)	6 (40%)	0	6 (40%)	15
Tagln-Cre; Ctnnb1$^{flox/WT}$ X *Ctnnb1*$^{flox/flox}$	44 (26%)	0	43 (25%)	83 (49%)	NE	NE	170
			**TOTAL**				**215**

NE, not expected with that specific breeding strategy.

Supplementary Table 2

Observed genotype distribution of mouse embryos at the indicated stages (E) used to determine the time of demise.

	Possible Genotypes from *Tagln-Cre; Ctnnb1*$^{flox/WT}$ X *Tagln-Cre; Ctnnb1*$^{flox/WT}$						
**Embryonic stage (E)**	*Ctnnb1*$^{flox/flox}$	*Tagln-Cre; Ctnnb1*$^{flox/flox}$	*Ctnnb1*$^{flox/WT}$	*Tagln-Cre; Ctnnb1*$^{flox/WT}$	*Ctnnb1*$^{WT/WT}$	*Tagln-Cre; Ctnnb1*$^{WT/WT}$	**Subtotal**
E9.5	0	3 (38%)	2 (25%)	2 (25%)	0	1 (12%)	8
E10.5	0	3 (38%)	1 (12%)	2 (25%)	0	2 (25%)	8
E11.5	2 (7%)	8 (29%)	2 (7%)	12 (43%)	1 (4%)	3 (11%)	28
E12.5	1 (6%)	5 (28%)	2 (11%)	5 (28%)	3 (17%)	2 (11%)	18
E13.5	1 (10%)	0	1 (10%)	5 (50%)	0	3 (30%)	10
E14.5	0	0	2 (33%)	3 (50%)	0	1 (17%)	6
E15.5	0	0	0	3 (60%)	0	2 (40%)	5
E17.5	0	0	0	1 (100%)	0	0	1
E19.5	1 (11%)	0	1 (11%)	4 (44%)	1 (11%)	2 (22%)	9
**TOTAL**							**93**

Supplementary Table 3

Observed genotype distribution of 21-day old pups that resulted from crosses of male *Ctnnb1*$^{dm/flox}$ with female *Tagln-Cre; Ctnnb1*$^{flox/WT}$ mice.

Genotype	Number of pups	%
Tagln-Cre; Ctnnb1$^{dm/flox}$ (*SMβC*^{dm})	0	0
Ctnnb1$^{dm/flox}$	14	23.7
Tagln-Cre; Ctnnb1$^{dm/WT}$	13	22.0
Ctnnb1$^{dm/WT}$	7	11.9
Tagln-Cre; Ctnnb1$^{flox/flox}$ (*SMβCKO*)	0	0
Ctnnb1$^{flox/flox}$	7	11.9
Tagln-Cre; Ctnnb1$^{flox/WT}$	8	13.6
Ctnnb1$^{flox/WT}$	10	16.9
TOTAL	59	100

Supplementary Table 4

Observed genotype distribution of 21-day old pups that resulted from crosses of male *Ctnnb1*$^{\Delta C/flox}$ with female *Tagln-Cre; Ctnnb1*$^{flox/WT}$ mice.

Genotype	Number of pups	%
Tagln-Cre;Ctnnb1$^{\Delta C/flox}$ (*SMβC*$^{\Delta C}$)	0	0
Ctnnb1$^{\Delta C/flox}$	20	24.1
Tagln-Cre; Ctnnb1$^{\Delta C/WT}$	9	10.8
Ctnnb1$^{\Delta C/WT}$	9	10.8
Tagln-Cre; Ctnnb1$^{flox/flox}$ (*SMβCKO)*	0	0
Ctnnb1$^{flox/flox}$	6	7.2
Tagln-Cre; Ctnnb1$^{flox/WT}$	19	22.9
Ctnnb1$^{flox/WT}$	20	24.1
TOTAL	83	100

Supplementary Table 5

Observed genotype distribution of 21-day old pups that resulted from crosses of male *Ctnnb1*$^{D164A/flox}$ with female *Tagln-Cre; Ctnnb1*$^{flox/WT}$ mice.

Genotype	Number of pups	%
Tagln-Cre; Ctnnb1$^{D164A/flox}$ (*SMβC*^{D164A})	4	4.0
Ctnnb1$^{D164A/flox}$	16	15.8
Tagln-Cre; Ctnnb1$^{D164A/WT}$	14	13.9
Ctnnb1$^{D164A/WT}$	25	24.8
Tagln-Cre; Ctnnb1$^{flox/flox}$ (*SMβCKO*)	0	0
Ctnnb1$^{flox/flox}$	11	10.9
Tagln-Cre;Ctnnb1$^{flox/WT}$	12	11.9
Ctnnb1$^{flox/WT}$	19	18.8
TOTAL	101	100
